# *Frag’n’Flow*: automated workflow for large-scale quantitative proteomics in high performance computing environments

**DOI:** 10.1186/s12859-025-06305-y

**Published:** 2026-01-04

**Authors:** Istvan Szepesi-Nagy, Roberta Borosta, Zoltan Szabo, Gabor E. Tusnady, Lorinc S. Pongor, Gergely Rona

**Affiliations:** 1 MTA-HUN-REN RCNS Lendulet “Momentum” DNA Repair Research Group, Institute of Molecular Life Sciences, HUN-REN Research Centre for Natural Sciences, Magyar Tudosok Korutja 2, Budapest, 1117 Hungary; 2 Semmelweis University Doctoral School, Budapest, 1085 Hungary; 3 Doctoral School of Biology, ELTE Eötvös Loránd University, Budapest, 1117 Hungary; 4 Department of Medical Chemistry, Albert Szent-Györgyi Medical School, University of Szeged, Szeged, 6720 Hungary; 5 Protein Bioinformatics Research Group, Institute of Molecular Life Sciences, HUN-REN Research Centre for Natural Sciences, Budapest, 1117 Hungary; 6 Department of Bioinformatics, Semmelweis University, Budapest, 1094 Hungary; 7 Cancer Genomics and Epigenetics Core Group, Hungarian Centre of Excellence for Molecular Medicine (HCEMM), Szeged, 6728 Hungary; 8 Department of Biochemistry and Molecular Pharmacology, NYU Grossman School of Medicine, New York, NY 10016 USA

**Keywords:** Quantitative proteomics, FragPipe, Nextflow, HPC, Mass spectrometry

## Abstract

**Background:**

Analysing large-scale mass spectrometry-based complex proteomics datasets often overwhelm desktop computational resources and require manual configuration for analysis. While *FragPipe* delivers rapid peptide identification across diverse sample preparation and acquisition modes (DDA, DIA, TMT), it remains challenging to deploy at scale.

**Results:**

We introduce *Frag’n’Flow*, a *Nextflow*‐based pipeline that encapsulates *FragPipe*, automates input manifest and workflow generation, manages tool dependencies and includes downstream data analysis options to enable reproducible, high‐performance analyses on HPC, cloud, and cluster environments. Benchmarking against other workflow-based solutions shows that our pipeline maintains quantitative accuracy and cuts runtime nearly in half on a typical DIA dataset of ~ 58 GB, while alleviating memory and I/O bottlenecks. We validate *Frag’n’Flow* results across three representative datasets, label-free DDA, DIA, and TMT, successfully recapitulating published biological signatures with minimal user intervention.

**Conclusions:**

By combining the sensitivity and speed of *FragPipe* with *Nextflow*’s orchestration, *Frag’n’Flow* enables the analysis of large‐scale proteomics data, empowering the scientific community, without extensive computation expertise, to extract new insights from existing MS datasets. *Frag’n’Flow* is available at: https://github.com/ronalabrcns/FragNFlow.

**Graphical abstract:**

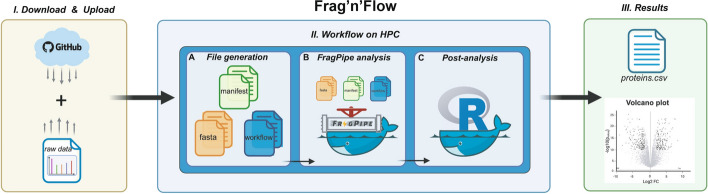

**Supplementary Information:**

The online version contains supplementary material available at 10.1186/s12859-025-06305-y.

## Background

Recent advances in mass spectrometry (MS) have transformed high-throughput proteomics, enabling the accurate and comprehensive analysis of thousands of proteins across diverse biological samples [1]. These advancements have broadened the scope of MS-based proteomics, from global proteome profiling to specialized applications essential for understanding cellular processes and disease mechanisms. The exponential growth of publicly available datasets has created a pressing need for advanced data processing tools to deepen our understanding of protein expression dynamics. Mass spectrometry is essential for capturing information that transcripts alone cannot provide, as it accounts for additional layers of regulation, such as post-translational modifications (PTMs). This underscores the importance of MS and the need to bridge the technological gap in processing large-scale proteomic datasets.

A variety of MS data analysis tools like *MaxQuant* [2], *Perseus* [3], *Skyline* [4], *DIA-NN* [5] or *FragPipe* (*FP*) [6–11] provide academic researchers with customizable, and accurate solutions that enable efficient processing of complex MS datasets. The increasing need for such tools is particularly evident in efforts to re-analyse existing studies to address new scientific questions. Among these tools, *FP* has gained widespread adoption due to its speed, accuracy, and compatibility with diverse acquisition and analysis formats. Its integrated tools support ultra-fast peptide identification and quantification, making it ideally suited for large-scale studies and PTM analysis [12, 13]. However, the scale and complexity of contemporary proteomic datasets frequently exceed the computational capabilities of standard laboratory setups. Challenges such as platform heterogeneity (*e.g.*, Windows, Linux, macOS), dependency management, and reproducibility across computational environments remain significant in large-scale computational proteomics.

In recent years, there has been a growing demand for platform-independent solutions to run and scale proteomics pipelines efficiently. Workflow orchestration platforms have emerged as powerful tools to modularize and automate traditionally monolithic proteomics applications, enabling their deployment across diverse large-scale computing systems, including high-performance computing (HPC) and cloud environments [14]. Although some earlier studies (such as *quantms*, *MHCquant* and *Galaxy-P*) have attempted to address the challenges of scalable proteomics workflows, the complexity of these solutions, coupled with the widespread adoption of *FP*, underscores the need for a dedicated workflow integration tailored specifically to *FragPipe* [15–17]. Currently, utilizing *FP* in HPC environments requires multiple manual interventions. Such manual steps include *fp-manifest* file generation, reference proteome setup via the graphical user interface (GUI), transferring workflow configurations to the HPC, and manually managing tool dependencies. Therefore, current deployment processes are often elaborate and time-consuming, limiting its accessibility and scalability in large-scale analyses.

To address these issues, we introduce *Frag’n’Flow*, an automated pipeline that integrates *FP* with *Nextflow*. *Frag’n’Flow* is a scalable, and high-performance solution, that leverages the full capabilities of the *FragPipe* toolkit while reducing user input and bias, thereby supporting adherence to FAIR (findability, accessibility, interoperability and reusability) principles [18]. *Nextflow* enhances *FP* by automating workflows across HPC, and cloud environments, ensuring portability and reproducibility [19]. Our integrated pipeline also streamlines downstream analyses, including differential expression, pathway mapping, and data visualization, while maintaining the convenience of deployment across diverse computing infrastructures for the broader research community.

## Implementation

### Development of *Frag’n’Flow*

For the development of *Frag’n’Flow*, we relied on the *Nextflow* workflow management framework (version 24.10.5). Various sub-workflow modules for file manipulation and script generation were implemented using GNU bash (version 5.2.21(1)-release) and R-base (R version 4.4.3). The MS raw file converter sub-module relies on *ProteoWizard’s MSConvert* tool, which is provided via DockerHub [20]. To utilize *FP*, we used the openly accessible *Docker* image [21] that *Nextflow* automatically retrieves and starts during execution. Although *Nextflow* supports multiple containerization methods, we used *Singularity* within our HPC environment to avoid requiring administrative privileges. For the fully customized mode, user-defined *FragPipe* workflows can be modified and executed through our pipeline, allowing for specific adjustments, such as changing the mass shift for the fixed modification applied during cysteine alkylation from iodoacetamide to MMTS (methyl methanethiosulfonate).

Unlike previous attempts to integrate *FP* into Unix-based systems, *Frag’n’Flow* allows the collection of licensed tools necessary for analysis (*MSFragger, IonQuant,* and *diaTracer* tools) directly from the official server, in compliance with their licensing terms (see Licensing information). Specifically, all required credentials and agreements are collected before execution. For detailed documentation, source code, available command line parameter options, and a minimum working example (MWE) please refer to *Frag’n’Flow*’s GitHub repository [22]. The MWE provides a step-by-step guidance covering dataset and reference proteome access, retrieval of licensed *FragPipe* tools with two-factor authentication procedures (2FA), initiation of the analysis, error handling strategies, and interpretation of the resulting differential protein expression outputs. This allows users to run *Frag’n’Flow* end-to-end within minutes, to quickly evaluate if the pipeline runs correctly.

### Downstream data analysis with *FragPipe-Analyst*

To facilitate downstream statistical analysis, we integrated the *FP-Analyst* module into the *Frag’n’Flow* workflow. This integration builds on the original *FragPipe-Analyst* webserver and enables automated downstream statistical analysis of protein or peptide quantification results. We employed the *FragPipeAnalystR* package (v1.0.5), a fully R-based implementation, encapsulated within a custom *Docker* container [23] to ensure reproducibility and consistency across computing environments. We utilized the Rocker project’s *r-base* Docker image (latest: 4.5.0) as a foundation image, then installed all necessary packages for *FragPipeAnalystR* through *BiocManager* (3.21) and *renv* (1.1.4) [24]. This way we ensured that the container includes all necessary dependencies and supports the latest versions of the underlying tools. Input files were processed in accordance with the specifications described in the original *FragPipe-Analyst* publication [24]. Briefly, quantification tables undergo normalization and missing value imputation (except for TMT workflows) before being subjected to differential expression (DE) analysis using the *limma* package [25]. *Limma* applies linear models with empirical Bayes moderation to estimate fold changes and statistical significance between conditions defined in the experimental annotation file. DE results are summarized through multiple diagnostic and interpretive visualizations. These include: principal component analysis plots; total protein/peptide bar plots; correlation heatmaps, and coefficient of variation plots for quality assessment. Additionally, volcano plots displaying significance (adjusted *p* values < 0.05 using the Benjamini–Hochberg method) and fold change (|log_2_FC|> 1); pairwise boxplots of differentially expressed proteins or genes; and GSEA visualizations are also generated using the *enrichR* package against the Hallmark or KEGG pathway databases. All statistical results, including normalized and imputed DE outputs, are saved in comma-separated value (CSV) format. Corresponding visualizations are compiled into a single PDF report for ease of interpretation and reporting.

### Licensing information

While the *FragPipe* computational platform is open-source and freely available, key tools required for its operation, namely *MSFragger, IonQuant*, and *diaTracer* for peptide identification and quantification, are only freely accessible for academic research and educational purposes. Therefore, the *Frag’n’Flow* pipeline integrates an automated download step to obtain and utilize these tools. To comply with the licensing terms and usage restrictions of the above-mentioned tools, *Frag’n’Flow* operates in a synchronous mode during initial runs to collect necessary user information. This includes the user's name, email address, and institutional affiliation, mirroring the GUI/Web-based download process from the original *MSFragger* webserver. Users must explicitly accept all license agreements provided by the Nesvizhskii Lab (University of Michigan) and complete a two-factor authentication process by submitting a code sent via email. Only after these steps are completed will the download of the required tools begin.

Importantly, *Frag’n’Flow* does not distribute, encapsulate, or otherwise share any licensed software. All software must be downloaded directly by the user via the authorized mechanisms. Since the “*MSFragger* + *IonQuant* + *diaTracer Suite*” is restricted to non-commercial academic use, the *Frag’n’Flow* pipeline also falls under the same licensing limitations and is intended exclusively for academic purposes.

*FragPipe* currently includes *DIA‑NN* version 1.8.2 due to licensing constraints that affect the deployment of later versions on cloud‑based or HPC infrastructures. While *Frag’n’Flow* allows users to download and use newer versions of *DIA‑NN* within the pipeline, users accessing version 1.9 or later must review and comply with the appropriate licensing terms, as certain restrictions may apply when running the software on systems not directly owned or operated by the user. For details, please refer to the *DIA‑NN* licensing terms.

### Hardware environments

For benchmark measurements and development, we utilized the institutional HPC in the HUN-REN Research Centre for Natural Sciences, administered and maintained internally by the institution. The HPC runs *Ubuntu 24.04.2 LTS,* SLURM workload manager for job submission (version 23.11.4) and Singularity containerization environment (4.1.1). For local measurements we relied on a desktop PC with Windows 11 OS (24H2) running WSL-based Ubuntu 24.04. The detailed specifications of the HPC environment and the laboratory PC specifications are listed in Table 1.

**Table 1:** Computation system specification used during benchmark analysis and development

Hardware	Institutional HPC environment	Laboratory PC
CPUs	Nodes with (32, 48, 64, 160) cores	16 cores
RAM	64–754 GB	96 GB
Data storage	33 TB ext4 RAID10	SSD 4 TB (Samsung 990 pro)
	28 TB NFS-mounted HDD	HDD 2 TB (Hitachi HUA72202)
Network	10 GB Ethernet	1 GB Ethernet
CPU architecture	Intel Xeon E5 family	AMD 9950X

### Benchmarking system requirements of *FragPipe*

To determine the optimal computational resources required for *FP* in large-scale proteomics workflows, we conducted a series of benchmark analyses focusing on random access memory (RAM) and CPU core utilization. Specifically, we assessed the memory demands of the *IonQuant* tool, a regularly utilized module in *FP*. For RAM benchmarking, we systematically increased the allocated memory (in gigabytes) relative to the number of input files analysed. This process was repeated iteratively until the analysis completed successfully using the minimum memory allocation on a laboratory workstation (*FragPipe* v22, Windows 11, AMD64, JAVA 17.0.10, date: February 25, 2025; Table 1), executed in GUI mode. Given the strong dependency of *TMT-Integrator* on *IonQuant*, which are key components of the *FragPipe* software suite for downstream quantification, memory profiling was conducted solely on *IonQuant*. Based on the empirical results, we interpolated the observed memory usage trends (*GraphPad*, third order cubic interpolation) to estimate theoretical RAM requirements for larger datasets that exceeded our available system memory. This interpolation enabled us to define memory thresholds beyond which local execution becomes infeasible.

To evaluate the minimum CPU requirements and runtime performance of individual tools within the LFQ-MBR workflow, we conducted systematic runtime benchmarks across varying file sizes using our institutional HPC cluster. Custom benchmarking scripts were developed for each utilized tool based on previous solutions [26]. We profiled the execution time of the following tools: *MSFragger*, *Percolator*, *Philosopher*, *IonQuant* [6, 9, 13, 27, 28], allowing us to assess the scaling behaviour of each module with respect to input data size and CPU resource allocation. For each tool, we defined the optimal configuration as the minimum number of CPU cores that achieved the shortest runtime or where additional cores yielded only diminishing returns (therefore the speedup plateaued). To capture the full computational profile of the analysis, we also included *MSConvert* and *DIA-NN* in the benchmarking alongside the other components.

### HDD, SSD storage device benchmarking

To assess the input/output performance characteristics of *FP*, we conducted a systematic benchmarking analysis by measuring processing time across datasets of varying sizes on two types of storage devices: a hard disk drive (HDD) and a solid-state disk (SSD). Two core *FP* workflows were evaluated: LFQ-MBR (for DDA) and DIA-SpecLib-Quant (for DIA) [29, 30]. Analyses were performed on our in-house laboratory PC workstation (Table 1).

For the DDA analysis, scalability and I/O efficiency were evaluated using incrementally larger datasets consisting of 10, 50, 200, and 350 *mzML*-format MS files from the MSBB study (detailed in Table 2 and Supplementary Files S1). For DIA analysis, five raw MS files in *.wiff* format were downloaded from the Answer ALS data portal and converted to *.mzML* (detailed in Table 2 and Supplementary File S1). To simulate increasing workload sizes, these files were duplicated in batches to create datasets representing 2×, 10×, 20×, and 40× file multiplications (Supplementary File S1). The resulting runtimes were recorded and compared between HDD and SSD configurations, providing insight into how storage media influence processing performance across workflows of differing computational demands.

**Table 2:** Summary of MS data used throughout the manuscript

Study name	Data ID	Source database	Data used for
Mount Sinai Brain Bank	syn20801227	AD knowledge portal	RAM, CPU, storage benchmarking
DIA MS analysis of primary breast cancers with no recurrence	PXD037428	PRIDE	CPU, storage benchmarking
Answer ALS	CASE-NEUXP595FFP CASE-NEUYP235ZLD CASE-NEUTU360YJY CASE-NEURF720KHA CASE-NEUGL543NJ1	Neuromine data portal	Storage benchmarking
Integrative analysis identifies key molecular signatures underlying neurodevelopmental deficits in FXS	PXD011630	PRIDE	Case study 1
DIA-MS analysis of 21 breast cancer tissues	PXD018830	PRIDE	Comparative analysis and Case study 2
Proteome and phosphoproteome data from the ccRCC tumors	PDC000127	CPTAC	Case study 3

### Data used for benchmark analysis

To support benchmark analysis, raw MS data were downloaded from three publicly accessible repositories: the AD Knowledge Portal [31], the PRIDE Proteomics Identifications Database [32], and the Answer ALS Neuromine portal [33] (Table 2, Supplementary File S1). These datasets were selected to represent both data-dependent acquisition (DDA) and data-independent acquisition (DIA) workflows (Supplementary Fig. S1A). For the DDA analysis, a total of 282 raw MS files were obtained from the Mount Sinai Brain Bank (MSBB) study [34], available via the AD Knowledge Portal. The samples were derived from post-mortem brain tissue (Brodmann area 10, anterior prefrontal cortex) of individuals diagnosed with Alzheimer's disease (AD). The total data volume for the DDA dataset was approximately 0.31 TB. For the DIA analysis, 21 primary breast cancer tissue samples were downloaded from PRIDE with accession number PXD037428, with a volume of 82.6 GB [35].

For comparative analysis with *quantms*, data were obtained from PRIDE (accession number: PXD018830), with a volume of 57.7 GB. The dataset consists of DIA mass spectrometry data derived from breast carcinoma tissue samples, resulting in a total of 25 DIA raw MS files including four controls [36]. This dataset was selected due to its compatibility with the *quantms* platform, which supports analysis of datasets annotated using the sample-to-data-relationship format (SDRF;*.sdrf.tsv*) [37]. The corresponding SDRF annotations for this dataset were publicly available and accessed via GitHub [38].

The UniProt human reference proteome (UP000005640, downloaded December 2, 2024) including reviewed and unreviewed sequences and isoforms was used for all database searches, unless otherwise specified. Protein grouping and filtering steps were applied to manage redundancy and ensure confident protein identifications.

### Data collection for case studies

To demonstrate exemplary use cases of the implemented analysis workflows, we utilized publicly available datasets (Table 2). For the DDA LFQ-MBR (label-free quantification with match-between-runs) workflow, raw proteomics data were obtained from the PRIDE repository (accession PXD011630). This dataset comprises of MS data derived from human pluripotent stem cell models of Fragile X syndrome (FXS). The dataset includes samples from four FXS-affected cells and four healthy controls [39]. For the DIA analysis workflow, we utilized the same dataset as in the quantms comparative analysis, described above [36]. For the tandem mass tag-based (TMT) quantification workflow, we analysed a 10-plex TMT dataset generated from clear cell renal cell carcinoma (ccRCC) samples, retrieved from the Clinical Proteomics Tumor Analysis Consortium (CPTAC; PDC ID: PDC000127; Run ID: 15CPTAC_CCRCC_Proteome_JHU_20180315). This dataset includes four primary tumor and four normal adjacent tissue (NAT) samples, selected according to the experimental design annotations provided by the CPTAC consortium [40].

## Results

### The I/O intensive nature of *FragPipe*

**Fig. 1:**
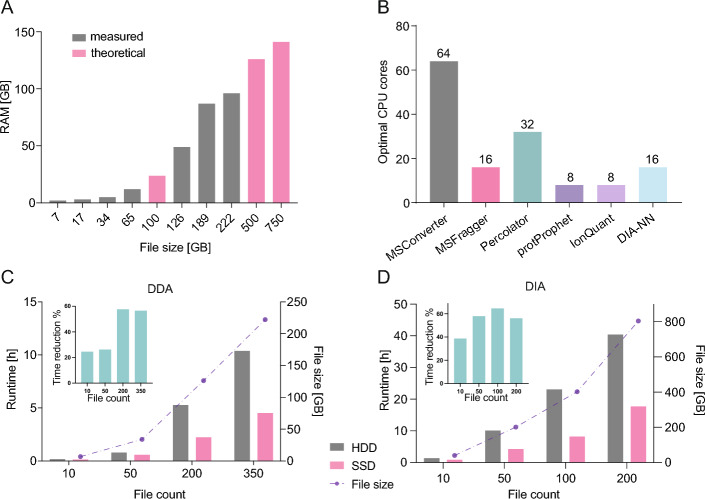
*FragPipe* shows strong monolithic functionalities through benchmarking. **A** Bar plots depict the minimum RAM requirements for the *IonQuant* tool. Grey indicates measured limits, while magenta represents theoretical limits using third order cubic interpolation for the indicated data size. **B** Bar plots indicate the optimal CPU requirements, measured as the minimum runtime for each individual *FP* tool, based on Supplementary Fig. S1B. **C**, **D** Runtime analysis of LFQ-MBR (DDA) and DIA-SpecLib-Gen (DIA) workflows respectively. Bar plots show analysis times using HDD (grey) or SSD (magenta) storage for the indicated file count and sizes. Insets highlight time reduction observed between HDD and SSD per file count across all analysis

Processing large proteomics datasets, such as when re-analysing raw data from repositories like PRIDE or MassIVE, requires handling files that often range from gigabytes to terabytes. Yet only a handful of MS-based analysis tools can manage data at this scale [41, 42]. *FragPipe* stands out as one of the most widely adopted platforms for MS-based proteomics, integrating a fast search engine, with rapid and sensitive peptide identification and quantification, across a variety of MS workflows. Its modular design, frequent updates, and broad support make *FP* particularly effective for large-scale studies, ensuring researchers to quickly extract biological insights from complex proteomic data. However, when using the GUI version of *FP* on a Windows system, the analysis of large datasets imposes considerable memory demands, often exceeding the capacity of standard laboratory workstations. For example, we observed that a dataset exceeding 228 GB failed to process successfully despite being run on a system with 96 GB of available RAM. To better understand these limitations, we systematically evaluated memory usage as a function of the number of input files using the *IonQuant* tool within *FP* GUI on our laboratory workstation (Table 1). We selected *IonQuant* for benchmarking because it is known to be a resource-intensive part of DDA data analysis and also serves as a core component of other widely used tools such as *TMT-Integrator*, which is commonly employed in TMT data analysis [26]. Based on the minimum RAM requirements, we extrapolated that processing a 0.75 TB dataset would require approximately 150 GB of RAM, well beyond the capabilities of typical laboratory workstations (Fig. 1A). While tools like *DIA-NN* are more memory-efficient for DIA [5], DDA and TMT continue to be widely used in large-scale proteomics studies, with a significant amount of publicly available data based on these methods.

To address the memory limitations encountered on standard laboratory workstations, we turned to the command-line interface (CLI) version of *FP* for Unix-based systems, enabling deployment on a HPC environment (Table 1). While this shift effectively resolved memory constraints, it also prompted a broader evaluation of system resource requirements, particularly CPU utilization. On our HPC system, we systematically evaluated CPU utilization across commonly used *FragPipe* workflows to better capture the system requirement landscape of the utilized individual tools. Through controlled benchmarking on large datasets (Supplementary Fig. S1A), we assessed how varying the number of available CPU cores influenced the runtime of individual tools within the *FP* pipeline. Our results indicate that most *FP* components do not substantially benefit from high CPU core counts. This suggests that performance gains plateau beyond modest parallelization, aligning with previous findings [26] showing that only customized *FP* modules can be effectively parallelized due to *FP’s* largely monolithic architecture (Fig. 1B, Supplementary Fig S1B and C).

While CPU scaling was not a major bottleneck compared to RAM limitations, we next sought to identify other performance-limiting factors. Given the multi-tool structure of quantitative proteomics workflows, we hypothesized that data input/output (I/O) operations may present a major constraint. These pipelines generate numerous intermediate files and require frequent reading and writing of large raw datasets, placing considerable demand on storage systems. To investigate this, we benchmarked runtime performance across different dataset sizes using both traditional HDD and SSD. Our findings highlight the monolithic nature of *FragPipe* and its strong dependency on storage performance. Switching from HDD to SSD led to substantial runtime reductions, with maximum decreases of 57% for DDA and 60% for DIA workflows (Fig. 1C and D, respectively). These results emphasize the importance of storage speed in optimizing *FragPipe* performance for large-scale proteomics analyses.

In summary, due to the substantial RAM demands and intensive data storage requirements of *FragPipe*, execution in a HPC environment can significantly enhance the efficiency of large-scale proteomics analyses. The availability of a containerized version of *FP*, operable via its headless CLI mode, allows integration into Unix systems. Although the containerized *FragPipe* can run on HPC environments, its reliance on GUI-based input generation and manual steps (*e.g.*, *fp-manifest* file generation, reference proteome setup, *workflow* transfer, and dependency management) limits its practicality for large-scale, command-line workflows. Our aim was to develop a pipeline to overcome these limitations, as detailed below.

### *Frag’n’Flow* overview

To enable seamless deployment of *FP* in HPC environments, we developed a *Nextflow*-based pipeline called *Frag’n’Flow*, providing a comprehensive and user-friendly solution for high-throughput proteomics workflows. It supports all major *FP* analysis modes, and leverages *Nextflow’s* flexibility for scalable, reproducible, and platform-independent execution. In brief, *Frag’n’Flow* automatically generates input files (manifest and workflow files), integrates the reference proteome (including decoy sequences), and selects the appropriate analysis workflow before initiating the main MS data processing and analysis. It automatically downloads all the necessary components of *FP* and is compatible with various tool versions. Additionally, built-in post-processing is streamlined through *FragPipe-Analyst*, which produces intuitive plots and summaries.

**Fig. 2:**
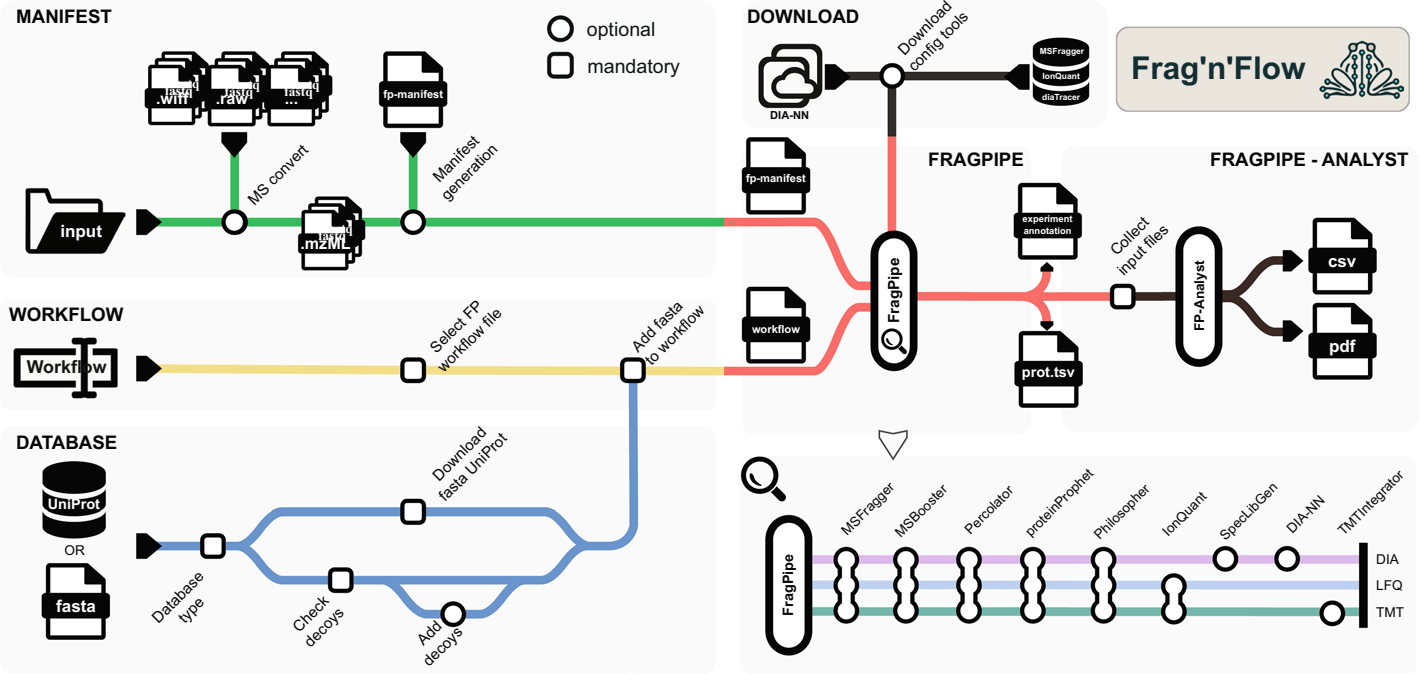
Overview of the *Frag’n’Flow* pipeline. Overview of the *Frag’n’Flow* workflow, composed of six modular sub-workflows (grey boxes), each representing a key functionality in the automated proteomics analysis pipeline. *Frag’n’Flow* supports all major predefined *FragPipe* (*FP*) analysis modes as well as custom user-defined configurations. Manifest *module* generates the *FP* input manifest file from a specified directory, with optional raw file conversion. Database *module* downloads the appropriate reference proteome and appends decoy sequences for target-decoy analysis. Workflow *module* selects and loads the *FP* analysis mode (*e.g.*, LFQ, TMT, DIA) to be executed. Download *module* retrieves necessary licensed tools (*e.g.*, *MSFragger*, *IonQuant*, *diaTracer*) in compliance with *FP* licensing agreements. *FragPipe core module* executes the mass spectrometry analysis using the manifest and workflow configuration files. *FragPipe-Analyst module* performs downstream statistical analysis and visualization, generating plots such as PCA, volcano plots, and heatmaps

Currently, initiating *FP* in CLI mode requires prior setup through its GUI. This involves manually specifying file paths, selecting data acquisition modes, manually defining the experimental design, and indicating replicate information. The GUI version then generates an *fp-manifest* file that consolidates these parameters. Additionally, the *workflow* file, used to define the analysis mode and integrate a path to the reference proteome with decoy sequences, must also be configured and saved through the GUI version. To exclude the need of the GUI interface and for the automation of these otherwise manual processes, we implemented four dedicated sub-modules within *Frag’n’Flow*. When executing our pipeline, two primary input files are generated to initiate the core proteomics analysis with *FP*: the manifest and workflow files, thereby enabling scalable and efficient large-scale analyses (Fig. 2). Additionally, to address limitations of previous solutions and to simplify deployability, we incorporated an automatic configuration module that downloads the necessary licensed tools for *FP* execution fully in line with *FragPipe’s* licensing terms.

### *Frag’n’Flow* pipeline

In the following sections, we detail the architecture, workflow design, and capabilities of *Frag’n’Flow* (Fig. 2). Further details about implementing *Frag’n’Flow* can be found at the project’s GitHub site [22].

*Manifest generation.* The manifest file is generated using a predefined file-naming convention, enabling automated creation of the required tab-separated input through a custom script. This approach allows for efficient management of input file paths directly on HPC systems. The manifest is automatically constructed from files located in the specified input directory. While *FP* natively supports *.raw* and *.mzML* file formats, we incorporated an optional preprocessing step to convert alternative formats, such as *.wiff* or proprietary *.raw*, into standardized *.mzML* files. When conversion is enabled, the manifest generation process automatically updates file paths to reference the converted *.mzML* files. As a core component of the analysis pipeline, the manifest generation module is both robust and flexible, supporting various naming conventions while ensuring compatibility with *FP’s* required input format. Alternatively, users may bypass the automated manifest generation by supplying a custom-defined manifest file.

*Database integration.* The database sub-module allows users to seamlessly incorporate a reference proteome FASTA file into the pipeline, either by providing an existing file or by downloading one directly from the UniProt web server. The latter option ensures convenient access to up-to-date reference proteomes. Regardless of the source, *Frag’n’Flow* automatically scans the FASTA file for the presence of decoy sequences. If none are found, reverse-order decoy sequences are appended by default, an essential step for generating a null model during peptide-spectrum match (PSM) evaluation. Additionally, as in *FP*, a list of common contaminants is added to the reference database. The finalized FASTA file, whether user-supplied or downloaded and modified, is then integrated into the workflow file corresponding to the selected analysis mode.

*Workflow selection. FragPipe* supports the analysis of multiple data types through a variety of tools, and to standardize these processes, pre-defined workflows are provided. These workflows refer to the configuration files within *FP* that specify toolsets and parameters for each type of analysis. All such workflow files are pre-included in the official containerized version of *FragPipe*, allowing users to select the appropriate analysis mode prior to execution. Originally, the workflow file with the already incorporated reference proteome path has to be saved and transferred from the GUI mode. To lighten this step for users working on non-GUI supported environments, *Frag’n’Flow* provides a sub-module that integrates the selected reference proteome into the chosen *FP* workflow configuration file, a critical step for the successful execution of the desired proteomics analysis. To enhance usability and accommodate varying needs based on sample preparation, instrument variance, or user preferences, users can also add custom workflow files in addition to the *FP*-based predefined ones.

*Configuration tools download module.* To simplify the setup processes of *FragPipe* on the desired system, we integrated the download processes for essential tools used by *FP*, which are subject to license agreements and would otherwise need to be manually downloaded using a GUI. These tools include *MSFragger*, *IonQuant*, and *diaTracer*, all of which are required for successfully running *FP*. The download sub-module requires users to initiate these downloads from the *MSFragger* server and explicitly accept the necessary license terms, fully in line with *FragPipe’s* licensing guidelines. This ensures that the most up-to-date versions of all configuration tools are retrieved, keeping the pipeline aligned with the latest *FP* releases and features.

With all four sub-modules in place, the necessary input files, configuration tools, and environment settings are automatically prepared, allowing *FP* to be launched seamlessly. *Frag’n’Flow* thus provides a fully automated setup for deploying *FP* in HPC or cloud environments, minimizing manual intervention and ensuring consistency across runs.

**Fig. 3:**
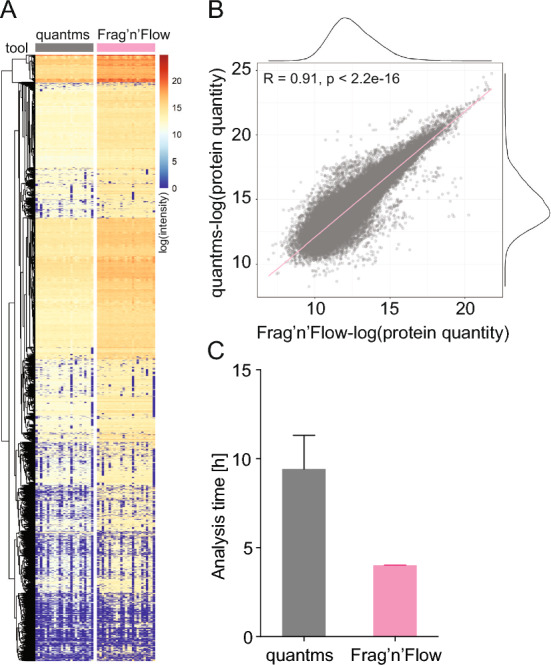
*Frag’n’Flow* achieves high concordance with faster runtimes than competing pipelines. **A** Heatmap representing log-normalized protein quantities identified by *quantms* and *Frag’n’Flow*. **B** Scatter plots with marginal densities depicting the correlation between *quantms* and *Frag’n’Flow* outputs, based on flattened vectors of the heatmap matrix in **A**. **C** Runtime performance comparison between *quantms* (grey) and *Frag’n’Flow* (magenta). The bar plot shows the total analysis time in hours. Error bars represent the standard deviation from three benchmark runs on the same dataset

*FragPipe execution.* This sub-module is designed to wait for all required input files, ensuring proper synchronization before proceeding. The integration of *FP* into the *Nextflow* environment is achieved by utilizing *FP’s* official containerized version which is maintained by the group of Alexey I. Nesvizhskii [21]. By default, *Frag'n'Flow* will download and use the *FragPipe* Docker image version 23.1, a version we have characterized and tested below. This version-pinning ensures long-term reproducibility and stability of *Frag'n'Flow*. Thanks to *FP’s* containerized architecture, users can easily download any desired version of the *FragPipe* container, ensuring access to new features and platform improvements while maintaining backward compatibility with previous tools. This setup enables the workflow to run *FP* in headless mode within the fetched container, referencing all required input files. With the help of the computational power of HPC environments, the *Frag’n’Flow* pipeline can run *FragPipe* workflows and produce the same output files as the desktop version, but with significantly faster performance (Fig. 3, Supplementary Fig. S2 and Supplementary File S2). Moreover, when used with an available workload manager, such as *SLURM*, *Frag’n’Flow* can efficiently distribute resource-intensive analyses across multiple compute nodes. To further optimize performance in such environments, *Nextflow’s* scratch and publishDir directive can be used to reduce I/O bottlenecks and provide flexible output handling tailored to the underlying storage system.

*Post-processing with FragPipe-Analyst. Frag’n’Flow* integrates a modular solution for proteomics data analysis, enabling users to directly process raw MS data with ease. To further enhance usability, we aimed to provide an intuitive and versatile visualization module that simplifies interpretation of the results. To achieve this, we integrated *FP-Analyst*, built on the *FragPipeAnalystR* package, into the *Frag’n’Flow* pipeline. This integration enables users to immediately visualize differential expression (DE) profiles generated by *FragPipe*, offering clear and informative insights through user-friendly plots and summaries.

When running the full *Frag’n’Flow* pipeline, required inputs such as the experimental annotation file and protein or peptide intensity data are automatically detected and passed to *FP-Analyst*. This automation eliminates the need for manual input in most cases, making it practical for large-scale analysis. The only exception is in TMT-based experiments, where users must manually define comparison groups in the annotation file, following standard *FP-Analyst* guidelines [24]. For added flexibility, *FP-Analyst* is also available in a standalone mode if users want to analyse previously processed datasets. In this configuration, users can provide the experimental design file and *FP* output manually, allowing integration into diverse workflows. *Frag’n’Flow* generates a comprehensive set of visual and statistical outputs to support both quality control and biological interpretation of DE analysis based on *limma*. For initial data assessment, it provides total protein/peptide abundance bar plots, principal component analysis (PCA) plots, correlation heatmaps, and coefficient of variation (CV) plots. PCA reveals sample clustering and potential batch effects, while heatmaps and CV plots evaluate reproducibility and variability. For DE results, *Frag’n’Flow* produces volcano plots that highlight significant changes in protein expression. Users may optionally generate pairwise boxplots to further explore specific DE features across conditions. To place the results in a functional context, gene set enrichment analysis (GSEA) is performed using the *enrichR* package. The resulting dot plots display enriched pathways from the Hallmark and KEGG databases, providing insight into the biological processes associated with the DE profiles. All outputs are saved as CSV files, and all visualizations are compiled into a single PDF report for ease of interpretation and sharing.

By integrating *FP-Analyst* into the *Frag’n’Flow* pipeline, our pipeline offers an end-to-end, modular solution that streamlines the analysis process from raw input to biologically interpretable output within a unified workflow.

### Comparative performance of *Frag’n’Flow*

To our knowledge, *quantms* [16] is the only currently available HPC-deployable proteomics workflow capable of analysing DDA, DIA, and TMT-based quantitative proteomics. We used *quantms* to benchmark the performance and output of *Frag’n’Flow* (Fig. 3, Supplementary Figs. S3 and S4). Both workflows were tested on a publicly available raw DIA dataset using default parameters and a standard *SLURM* workload manager. DIA data were selected because of their increasing adoption in the field and the cross-platform comparability of *DIA-NN* outputs. Additionally, while *quantms* relies on *MS-GF* + or *Comet* for peptide identification in DDA workflows, *FragPipe* uses *MSFragger*, reflecting a fundamental difference in identification strategies between these pipelines.

We collected runtime metrics for the full workflow, including critical steps such as data download, file conversion, PSM, spectral library generation, and *DIA-NN*-based quantification. Importantly, protein intensity values showed a strong correlation (R = 0.91, *p* = 2.2 × 10^–16^) between *Frag’n’Flow* and *quantms*, both at the individual sample level and across the entire protein intensity matrix (Supplementary Figs. S3; Fig. 3A). Matrix transformation further confirmed that both workflows produced similarly structured datasets (Fig. 3B). These results support the conclusion that *Frag’n’Flow* provides comparable output to *quantms*.

In terms of computational performance, the *Frag’n’Flow* pipeline completed the full analysis in around half the time required by *quantms* on a dataset of ~ 58 GB (Fig. 3C). Both pipelines exhibited similar execution times for most steps, with the exception of the average raw data conversion time (Supplementary Fig. S4) and the spectral library generation. The latter significantly extended the total runtime of *quantms* (Supplementary Fig. S4E). In summary, *Frag’n’Flow* matched *quantms* in analytical performance and biological interpretability, while delivering faster spectral library generation.

### Case studies demonstrating *Frag’n’Flow*

**Fig. 4:**
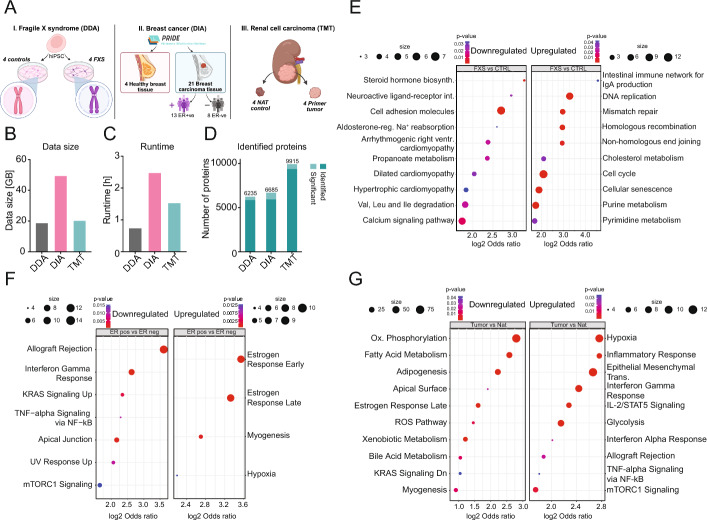
Case studies of *Frag’n’Flow* analysing all major data acquisition data types (DDA, DIA and TMT). **A** Schematics show the experimental design of each case study used. **B** Bar plot indicates the size of the analysed datasets. **C** Bar plot shows the total analysis runtime in hours for each of the datasets. **D** Bar plot shows the number of total identified proteins along with the ones that showed significant differential expression for each of the datasets. **E** Panels show the GSEA results of the FXS case study (DDA data) based on KEGG database, highlighting the upregulated (right) and downregulated (left) pathways. **F** Panels show the GSEA results of the breast cancer case study (DIA data) using Hallmark database, highlighting the upregulated (right) and downregulated (left) pathways. **G** Panels show the GSEA results of the ccRCC case study (TMT 10-plex) using Hallmark database, highlighting the upregulated (right) and downregulated (left) pathways

We present three representative case studies, each illustrating one of the major MS based quantitative analytical methods (Fig. 4A), to demonstrate *Frag’n’Flow’s* runtime, analysis quality, and concordance with published results. When comparing to published data, discrepancies can arise from various technical factors, including library generation, normalization, imputation, PTM settings, enzyme specificity, false discovery rate (FDR) calculation methods, and threshold values. Therefore, we primarily focused on comparing GSEA outputs to assess whether similar biologically relevant trends were observed.

*Fragile X Syndrome hiPSC Data Set.* In our first example, we reanalysed a label-free, DDA dataset of human iPSC-derived neurons from Fragile X Syndrome (FXS) patients. Using FP’s LFQ-MBR [29] workflow and default parameters, we processed raw files through the *MSConvert*, *FP*, and *FP-Analyst* modules of *Frag’n’Flow*. For DE analysis, we compared FXS and healthy, control samples. The full pipeline processed 18.6 GB of data in 44 min, identifying 385 significantly differentially expressed proteins (Fig. 4B–D). Importantly, our results reproduced key protein abundance trends reported in the original study. For example, GPRIN3, CNTN1, CNTNAP2 and KIF5C proteins, that have been previously associated with FXS pathophysiology were found to be significantly downregulated in FXS (Supplementary Data S1) [39]. To further validate our results, we conducted a KEGG pathway analysis from the original article using *g:Profiler* [43] to compare these results with the GSEA outputs originating from *Frag’n’Flow*. We identified significant enrichment of DNA repair-related pathways, including mismatch repair (MMR), homologous recombination (HR) or non-homologous end-joining (NHEJ), in line with the original study (Fig. 4E and Supplementary Data S1).

*Human Breast Carcinoma Data Set.* Next, we analysed a publicly available breast cancer DIA dataset using the DIA-SpecLib-Quant [30] workflow. The dataset includes proteomics data of estrogen receptor (ER) positive and negative tumour samples [36]. We used *Frag’n’Flow* and the *FP-Analyst* embedded within our pipeline to compare the DE profiles of ER-positive and ER-negative samples with those reported in the source publication. The full dataset (49.2 GB) was processed in 2 h and 27 min, resulting in 755 differentially expressed proteins (Fig. 4B–D). GSEA was performed against the Hallmark database, successfully reproducing key biological findings of the original study, including significant enrichment of estrogen response pathways as well as significant downregulation of allograft rejection and interferon gamma response pathways (Fig. 4F and Supplementary Data S2). These results align with the known molecular distinction between ER-positive and ER-negative breast cancers.

*Clear Cell Renal Cell Carcinoma Data Set.* We also applied *Frag’n’Flow* to a TMT 10-plex dataset from CPTAC data base, focusing on clear cell renal cell carcinoma (ccRCC) [40]. TMT data, while compact and multiplexed, requires manual specification of experimental groups. As such, this is the only workflow mode where pipeline execution is divided to allow modification of the experimental annotation file prior to DE analysis. For this case study, we analysed a single TMT plex comprising four tumour and four adjacent normal (NAT) tissue samples. The 20.1 GB dataset was processed using the *MSConvert*, *FP* and *FP-Analyst* modules completing in approximately 1.5 h, identifying 561 differentially expressed proteins (Fig. 4B–D). Following this, we manually updated the experimental annotation file to define the tumour versus NAT comparison and executed the *FP-Analyst* module accordingly. GSEA, using the Hallmark database, reproduced key findings from the original CPTAC publication. Specifically, we observed strong upregulation of hypoxia and inflammatory response pathways (Fig. 4G), both of which are hallmarks of the ccRCC tumor microenvironment [40]. Additionally, our analysis confirmed the downregulation of oxidative phosphorylation processes, with key genes such as NDUFV2 and COX6C showing significant decreases in expression (Supplementary Data S3), consistent with metabolic reprogramming in ccRCC. Additionally, our GSEA results revealed an enhanced inflammatory response signature, marked by increased expression of genes associated with Wnt signalling (ICAM1, HIF1A), a known master regulator of inflammation, tumour progression, and metastasis [44, 45]. This represents a novel observation that extends previous findings and highlights the additional biological insight enabled by *Frag’n’Flow*.

These three case studies collectively demonstrate the effectiveness, and reproducibility of *Frag’n’Flow* across all major quantitative proteomics analytical formats: DDA, DIA, and TMT. In each case, our pipeline successfully reanalysed publicly available datasets in an HPC environment, reproducing key biological findings reported in the original publications. These examples highlight the ability to deliver fast, reliable, and biologically meaningful results across diverse experimental setups by reanalysing publicly available data using an easily deployable and scalable *FragPipe* pipeline on HPC systems.

## Discussion

Advances in mass spectrometry-based proteomics have markedly expanded our ability to interrogate complex biological systems, yet the sheer scale of modern datasets often outpaces the computational resources and expertise available in many laboratories. *Frag’n’Flow* addresses this gap by combining *FragPipe’s* highly sensitive peptide identification engine with *Nextflow’s* mature workflow orchestration, resulting in a pipeline that is both high-performance and fully automated. This integration enables a reproducible platform and user independent solution that is HPC- and cloud-ready. In contrast to many existing workflows that require manual intervention, GUI input or lack scalability, the *Frag’n’Flow* pipeline streamlines complex analyses with minimal user input while maintaining reproducibility and flexibility across different computing environments following FAIR [18] recommendations.

Our systematic benchmarking confirmed that conventional *FragPipe* GUI deployments quickly exceed the memory capacities of typical desktop workstations, and that only modest performance gains can be achieved through core‐count scaling. In large-scale HPC deployments, additional performance challenges arise from *FragPipe’s* high I/O demands, which can lead to bottlenecks under concurrency. Users can set up their HPC environment to use the scratch directive in *Nextflow*, that enables intermediate files to be written to a local working directory before being transferred back to persistent storage, thereby alleviating metadata pressure and contention on shared filesystems. Furthermore, users can also configure the publishDir functionality to copy or move results after task completion, offering flexible output handling tailored to the storage characteristics of the target system. We recommend that users also consult the *Nextflow* documentation for guidance and best practices, as it provides recommendations tailored to different HPC environments.

*Frag’n’Flow* provides a containerized command-line interface for *FragPipe* and integrates our novel developments, including automated manifest creation, reference database setup, workflow selection, built-in tool downloads (in compliance with licensing agreements), and downstream data analysis options. By eliminating the need for manual configuration, *Frag’n’Flow* delivers an efficient and user-friendly deployment experience. At the same time, it supports user-defined workflows and settings, offering flexibility for in-depth customization and future adaptability, including simplified on-the-fly workflow modifications. Planned developments include support for custom contaminant lists in reference databases and expanded options for parameterizing existing workflows.

Compared to the *quantms* pipeline, *Frag’n’Flow* achieved comparable quantitation accuracy while completing end-to-end analyses in less than half the runtime. This performance gain can be attributed to the effective library-building process and optimized input/output operations on high-performance filesystems. Nonetheless, it is important to note that *quantms* is fully open-source and free of licensing restrictions, making it a strong alternative, particularly for use beyond academic settings. Our three real-world case studies, spanning all major MS-based quantitative analytical methods (DDA, DIA, and TMT) demonstrate successful re-analysis of data with the *Frag’n’Flow* pipeline. The results accurately reproduce established biological signatures, including published differential expression and pathway enrichment results, while also uncovering additional insights with minimal manual input.

## Conclusions

In conclusion, our workflow enables researchers to analyse the rapidly growing large-scale raw proteomics datasets in repositories such as PRIDE and MassIVE by leveraging HPC resources to process data ranging from hundreds of gigabytes to terabytes. We hereby present *Frag’n’Flow* a novel pipeline to process large-scale proteomics datasets. *Frag’n’Flow* transforms *FragPipe* from a powerful yet desktop‐oriented application into a robust, reproducible, and user‐friendly HPC based pipeline capable of meeting the demands of modern, large‐scale proteomics. By marrying the sensitivity and speed of *FragPipe* with *Nextflow’s* orchestration and modularity, our pipeline enables rapid, reproducible extraction of biological insights from complex MS datasets, thereby accelerating discovery across diverse biomedical research areas.

### Availability and requirements

Project name: Frag’n’Flow

Project home page: https://github.com/ronalabrcns/FragNFlow

Operating system(s): Platform independent, Linux, Windows, Mac

Programming language: Nextflow (Groovy), Bash, R

Other requirements: DSL2, Nextflow v22.03.0-edge+ 

License: MIT license

Any restrictions to use by non-academics: License needed. For detailed licensing information for FragPipe, it’s modules and DIA-NN, please refer to the “Licensing information” section.

## Supplementary Information

Additional file 1: **Data S1.** Contains results and output files from the DDA case study. Table S1. Comma-separated values (CSV file) includes the identified protein intensities along with the differential expression analysis results. Output S1. (PDF file) is the FP-Analyst generated report with all corresponding visualizations and outputs.

Additional file 2: **Data S2.** Contains the results and output files from the DIA case study. Table S2. (CSV file) includes the identified protein intensities and differential expression analysis results, based on the data-independent acquisition workflow. Output S2. (PDF file) is the FP-Analyst generated report with all corresponding visualizations and outputs.

Additional file 3. **Data S3.** Contains the results and output files from the TMT case study. Table S3. (CSV file) provides quantified protein intensities and differential expression analysis results obtained from the tandem mass tag (TMT) experiments. Output S3. (PDF file) is the FP-Analyst generated report with all corresponding visualizations and outputs.

Additional file 4: **Figure S1.** Benchmark measurements of the individual tools of *FragPipe*. **A** Schematics show the experimental design of the case studies used for the benchmarks. **B** Runtime analysis of individual FP tools with different CPU settings. Error bars indicate standard deviation from 3 independent runs. **C** Speedup values calculated from the runtime analysis in (B). Error bars indicate standard deviation from 3 independent runs. The dashed line shows the overall speedup trend, while the orange curves represent speedup plateaus corresponding to the local minima observed in (B).

Additional file 5: **Figure S2.** Correlation of peptide (left) and protein (right) intensities between *FragPipe *analysis methods. Scatter plots comparing peptide and protein intensities obtained from the FragPipe GUI and the *Frag’n’Flow* pipeline. Peptides and proteins with zero intensity were excluded. Trendlines and Pearson correlation coefficients (R-values) are shown to illustrate the degree of agreement between the two methods.

Additional file 6: **Figure S3.** Correlation of normalized protein intensities between samples. Scatter plots comparing log-normalized protein intensities from 25 DIA samples, analysed using *quantms *and* Frag’n’Flow* workflows. Each point represents a protein intensity measurement detected in both workflows. Only proteins identified and quantified in both pipelines were plotted. Trendlines and Pearson correlation coefficients (R-values) are shown to illustrate the degree of agreement between the two methods.

Additional file 7: **Figure S4.** Comparative analysis of quantms and *Frag’n’Flow*. **A** Schematics show the experimental design of the case study (DIA dataset) used for the benchmark. **B** Comparison of total download time for retrieving raw MS files from the PRIDE FTP server in hours. Error bars represent the standard deviation from three benchmark runs on the same dataset. **C** Average raw MS file conversion time per file during workflow execution in minutes. Error bars represent the standard deviation from three benchmark runs on the same dataset. **D** Comparison of total time needed for peptide spectrum in each of the workflows in hours. Error bars represent the standard deviation from three benchmark runs on the same dataset. **E** Comparison of total time needed for spectral library generation in hours. Error bars represent the standard deviation from three benchmark runs on the same dataset. **F** Comparison of total runtime of *DIA-NN* modules in both workflows in minutes. Error bars represent the standard deviation from three benchmark runs on the same dataset.

Additional file 8: **File S1.** Detailed description of data used from the AALS and MSBB repositories in Fig. 1.

Additional file 9: **File S2.** (**A**: sheets 1 to 4) Comparison of peptide and protein level outputs generated using the *FragPipe* GUI and the *Nextflow*-based *Frag’n’Flow* pipeline. (**B**: sheets 5 to 6) Protein level intensity matrices from *Frag’n’Flow* and *quantms*, used in benchmarking analysis.

## Data Availability

All data generated or analysed during this study are included in this published article and its supplementary information files. Raw MS data used throughout the manuscript are publicly accessible and listed in Table 2. Details on data access and the raw data used for the benchmark analysis of the AALS and MSBB portals are provided in Supplementary File S1. The source-code, instructions, and a minimal working example of Frag’n’Flow are publicly available through the GitHub repository [22]. Any further information on datasets used and/or analysed during the current study are available from the corresponding author upon request.

## Abbreviations

2FA: Two-factor authentication
ccRCC: Clear cell renal cell carcinoma
CLI: Command line interface
CSV: Comma-separated value
CV: Coefficient of variance
DDA: Data-dependent acquisition
DE: Differential expression
DIA: Data-independent acquisition
ER: Estrogen receptor
FDR: False discovery rate
FP: FragPipe
FXS: Fragile X syndrome
GSEA: Gene set enrichment analysis
GUI: Graphical user interface
HDD: Hard disk drive
HPC: High performance computing
HR: Homologous recombination
I/O: Input/output
LFQ-MBR: Label-free quantification with match-between-runs
MMR: Mismatch repair
MS: Mass spectrometry
MSBB: Mount Sinai Brain Bank
MWE: Minimum working example
NAT: Normal adjacent tissue
NHEJ: Non-homologous end joining
PCA: Principal component analysis
PSM: Peptide spectrum match
PTMS: Post-translational modifications
RAM: Random access memory
SDRF: Sample-to-data-relationship format
SSD: Solid-state disk
TMT: Tandem mass tag
