# Supplementary material for: Frag’n’Flow: automated workflow for large-scale quantitative proteomics in high performance computing environments
Source: BMC Bioinformatics. 2026 Jan 4;27:18. doi: 10.1186/s12859-025-06305-y (PMC12828970; doi:10.1186/s12859-025-06305-y)
Supplement: Supplementary file 1 — Additional file 1: Data S1. Contains results and output files from the DDA case study. Table S1. Comma-separated values (CSV file) includes the identified protein intensities along with the differential expression analysis results. Output S1. (PDF file) is the FP-Analyst generated report with all corresponding visualizations and outputs. [file 12859_2025_6305_MOESM1_ESM.zip › Data S1/Output S1.pdf]

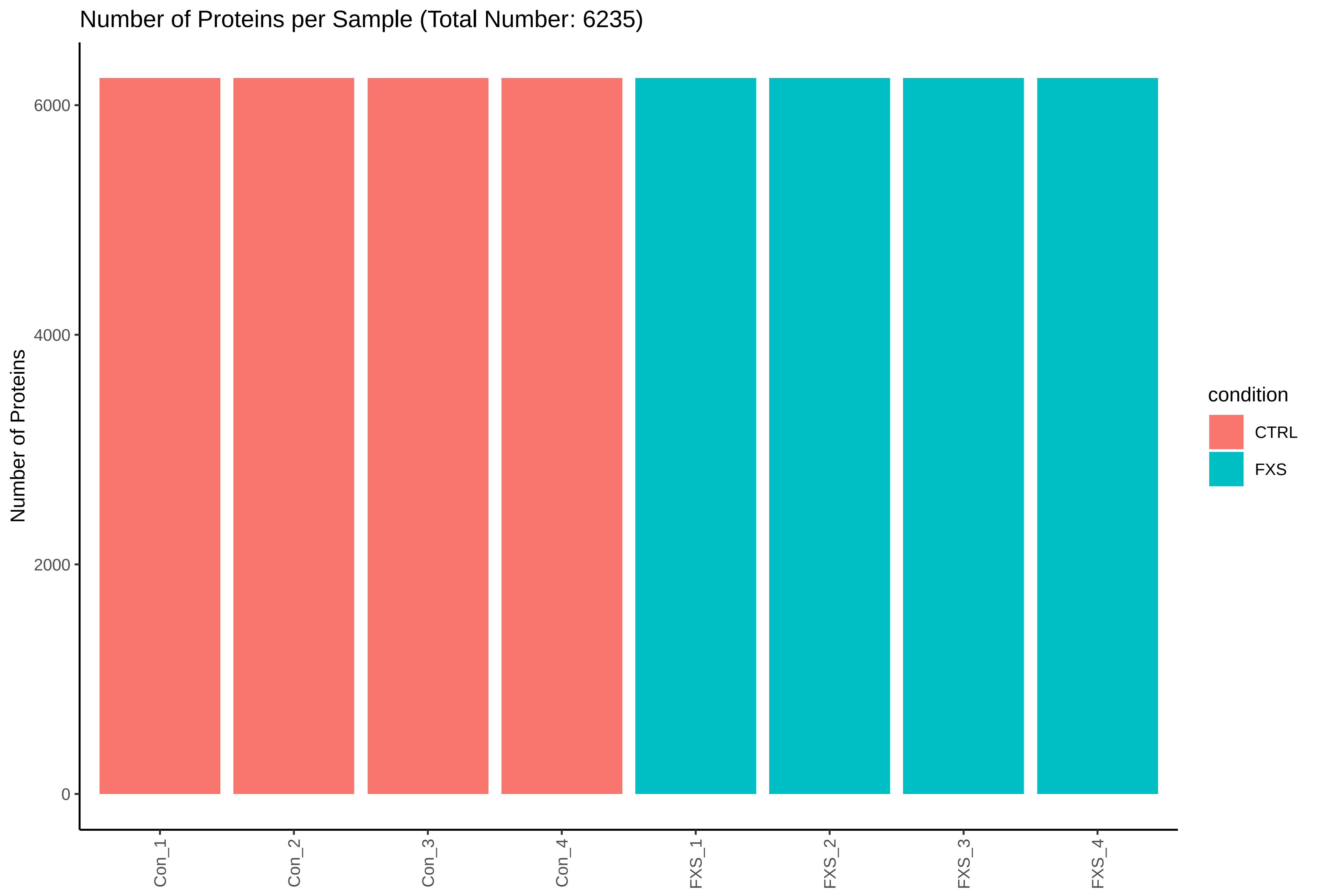
Number of Proteins per Sample (Total Number: 6235)
Number of Proteins
6000
4000
2000
0
Con_1
Con_2
Con_3
Con_4
FXS_1
FXS_2
FXS_3
FXS_4
condition
CTRL
FXS

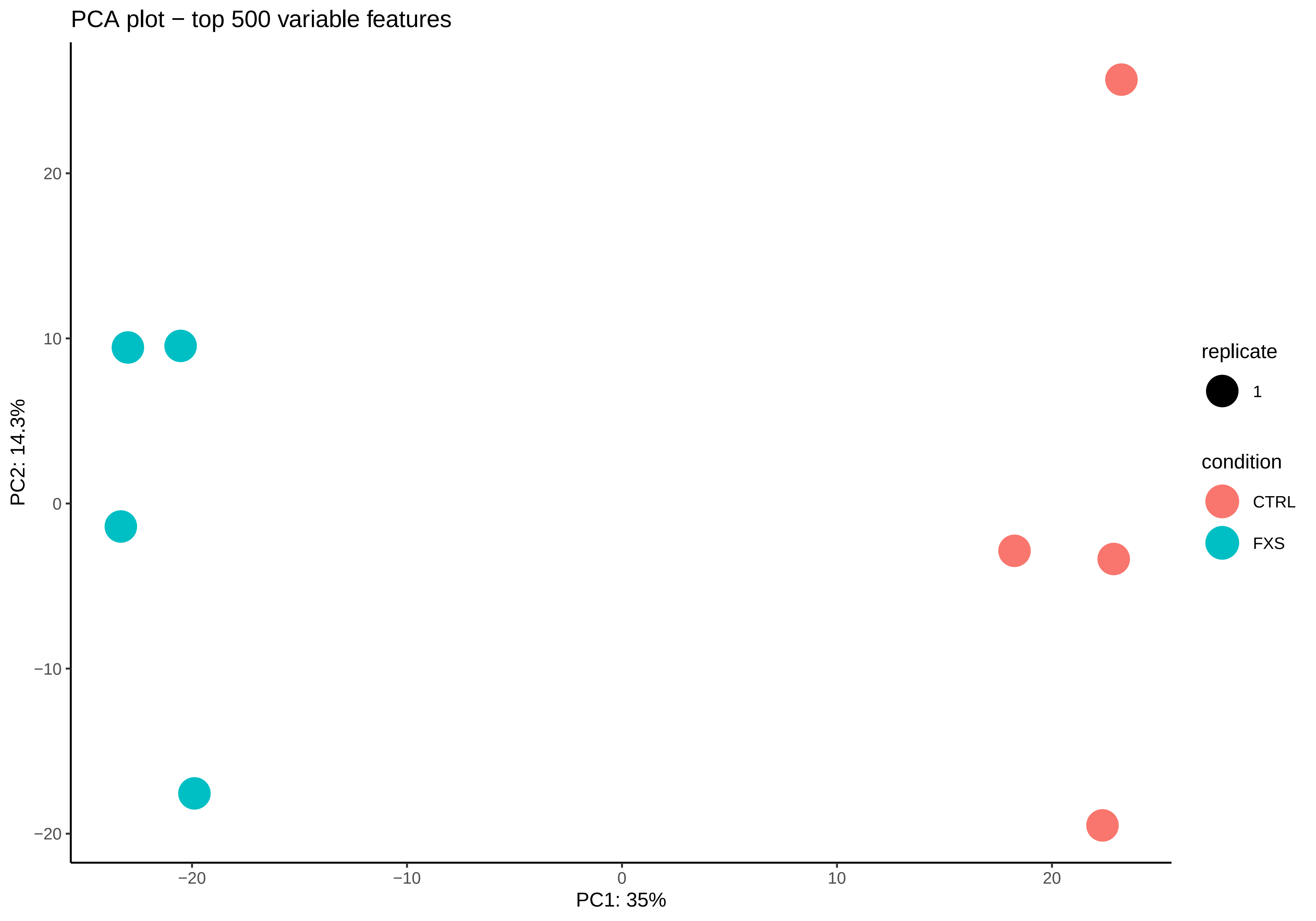
PCA plot − top 500 variable features
PC1: 35%
PC2: 14.3%
−20
−10
0
10
20
replicate
1
condition
CTRL
FXS

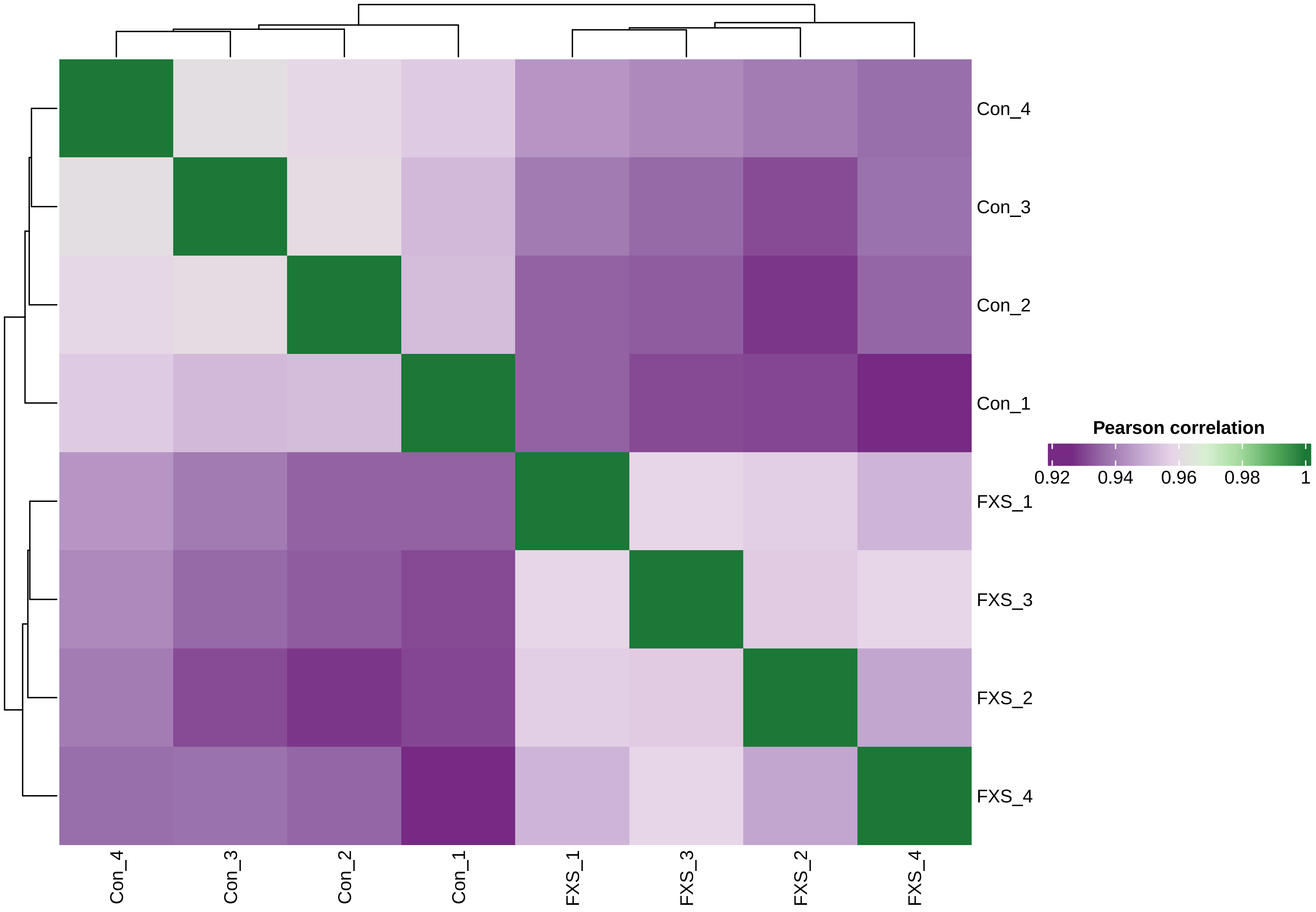

Con_4
Con_3
Con_2
Con_1
FXS_1
FXS_3
FXS_2
FXS_4
Pearson correlation
0.92
0.94
0.96
0.98
1

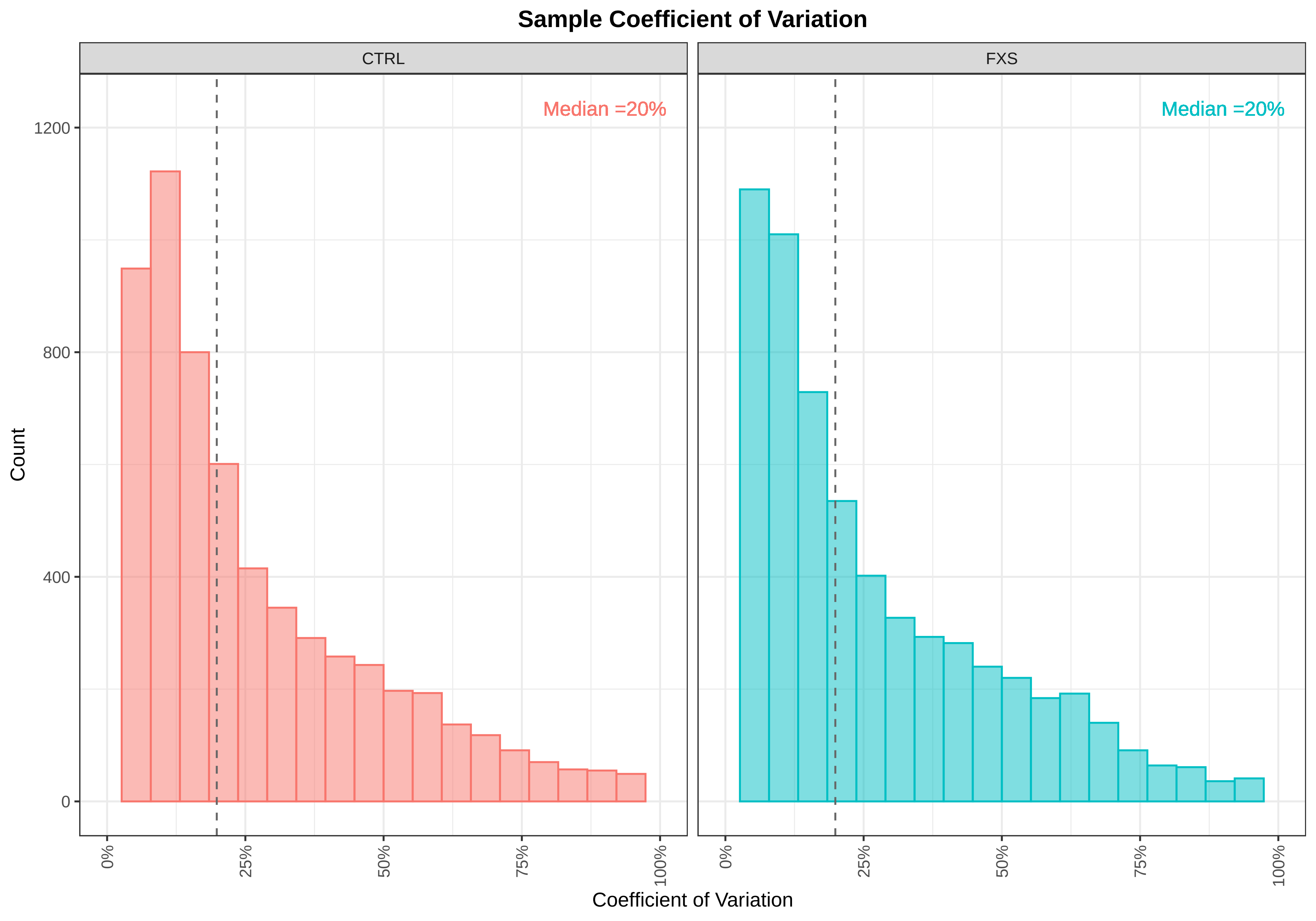

Sample Coefficient of Variation
CTRL
FXS
Median =20%
Median =20%
Count
1200
800
400
0
0%
25%
50%
75%
100%
Coefficient of Variation

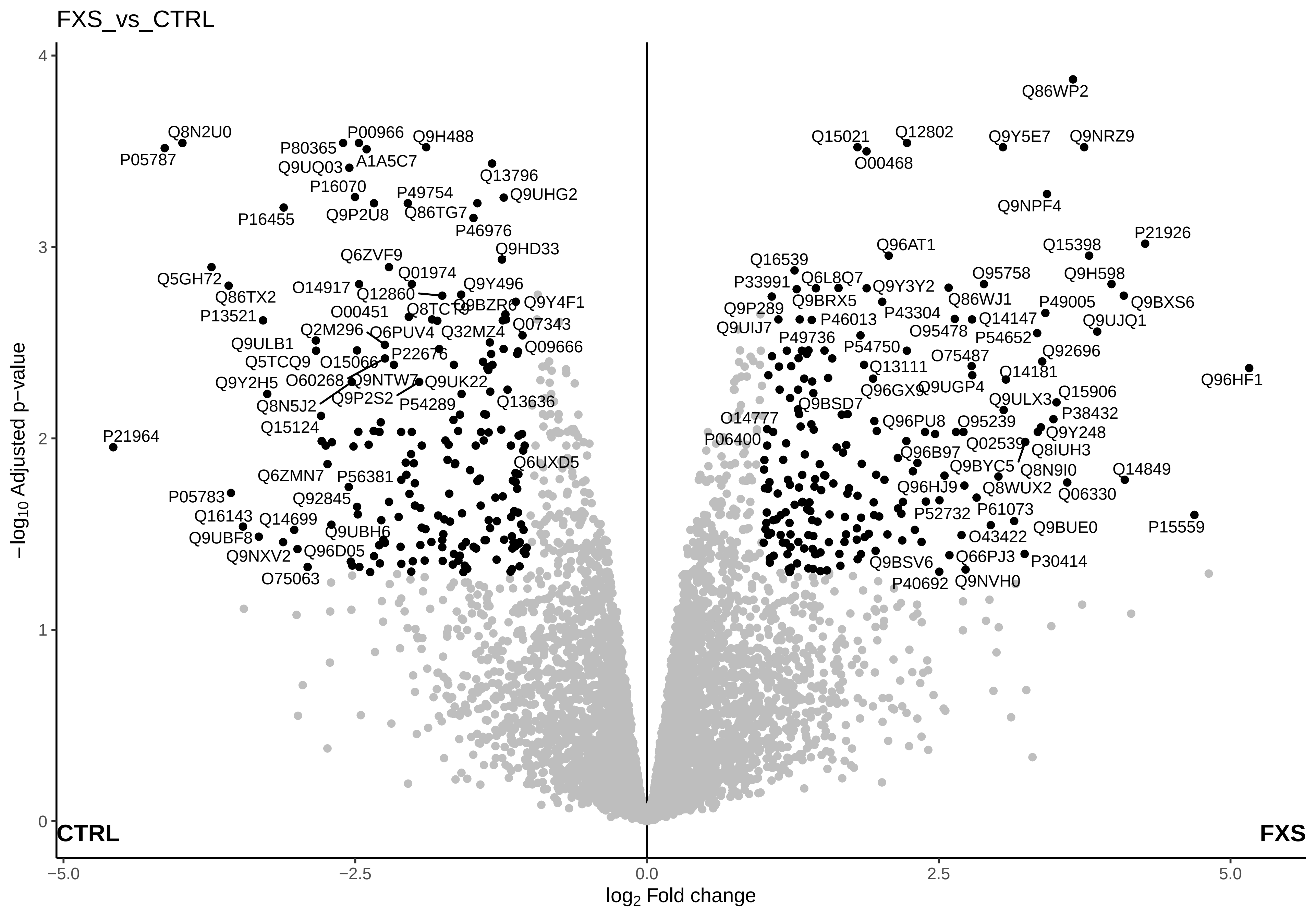

FXS_vs_CTRL
−log10 Adjusted p−value
log2 Fold change
CTRL
FXS
−5.0
−2.5
0.0
2.5
5.0
0
1
2
3
4
Q8N2U0
P05787
P00966
P80365
Q9H488
A1A5C7
Q9UQ03
P16070
P49754
Q13796
Q9UHG2
P16455
Q9P2U8
Q86TG7
P46976
Q6ZVF9
Q9HD33
Q5GH72
Q01974
O14917
Q9Y496
Q12860
Q86TX2
Q9BZR6
Q9Y4F1
P13521
O00451
Q8TCT0
Q2M296
Q6PUV4
Q32MZ4
Q07343
Q9ULB1
P22676
Q09666
Q5TCQ9
O15066
O60268
Q9NTW7
Q9UK22
Q9Y2H5
Q9P2S2
P54289
Q13636
Q8N5J2
Q15124
P21964
Q6UXD5
Q6ZMN7
P56381
P05783
Q92845
Q16143
Q14699
Q9UBH6
Q9UBF8
Q96D05
Q9NXV2
O75063
Q86WP2
Q15021
Q12802
Q9Y5E7
Q9NRZ9
O00468
Q9NPF4
P21926
Q96AT1
Q15398
Q16539
Q6L8Q7
Q95758
Q9H598
P33991
Q9Y3Y2
Q86WJ1
Q9BXS6
Q9BRX5
P49005
Q9P289
P43304
P46013
Q14147
Q9UJQ1
Q9UIJ7
O95478
P54652
P49736
P54750
Q92696
O75487
Q13111
Q14181
Q96GX9
Q9UGP4
Q96HF1
Q9BSD7
Q15906
Q9ULX3
O14777
P38432
Q96PU8
O95239
P06400
Q9Y248
Q02539
Q8IUH3
Q96B97
Q9BYC5
Q8N9I0
Q14849
Q96HJ9
Q8WUX2
Q06330
P52732
P61073
Q9BUE0
P15559
O43422
Q9BSV6
Q66PJ3
P30414
P40692
Q9NVH0

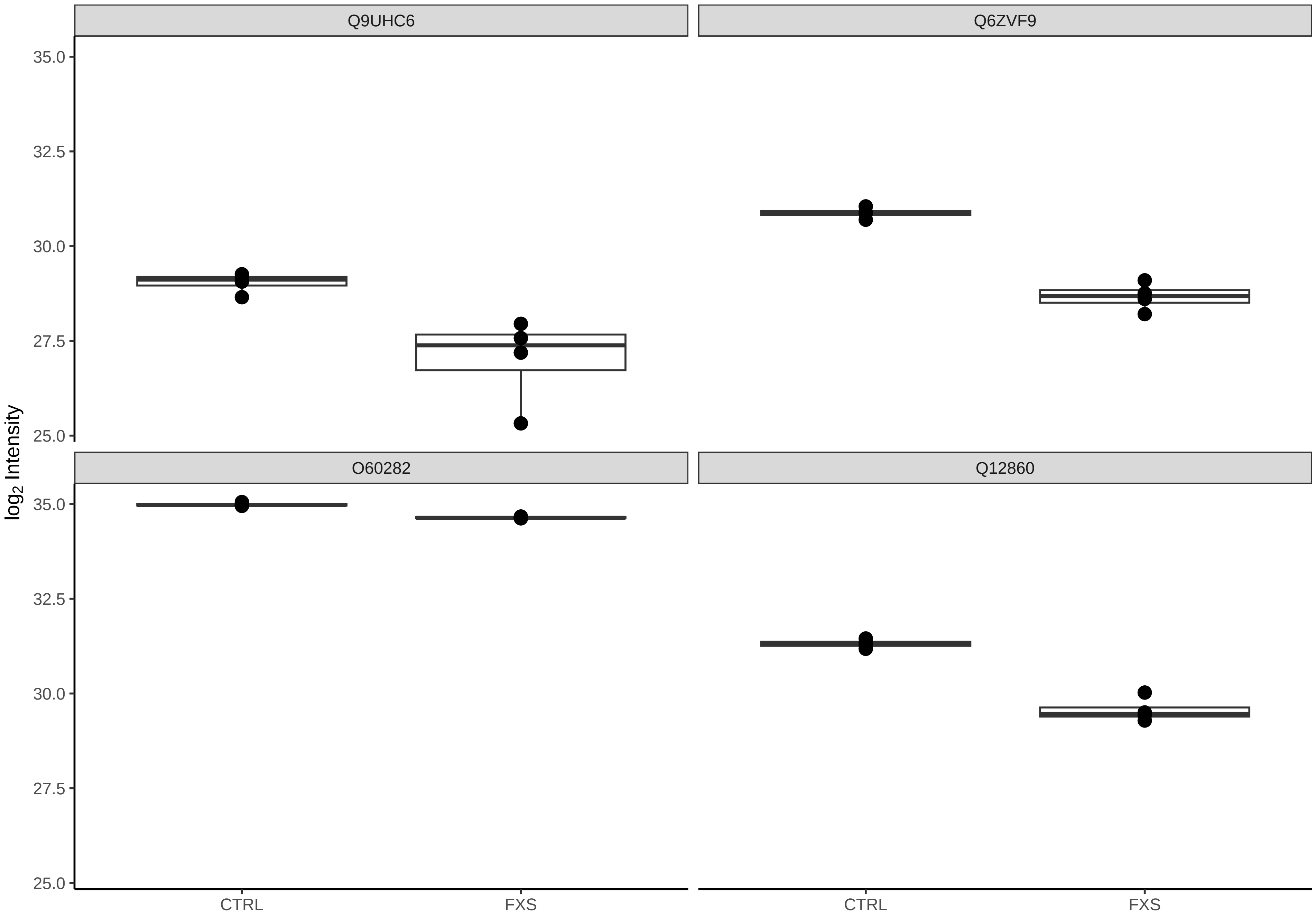

Q9UHC6
Q6ZVF9
O60282
Q12860
log2 Intensity
35.0
32.5
30.0
27.5
25.0
CTRL
FXS

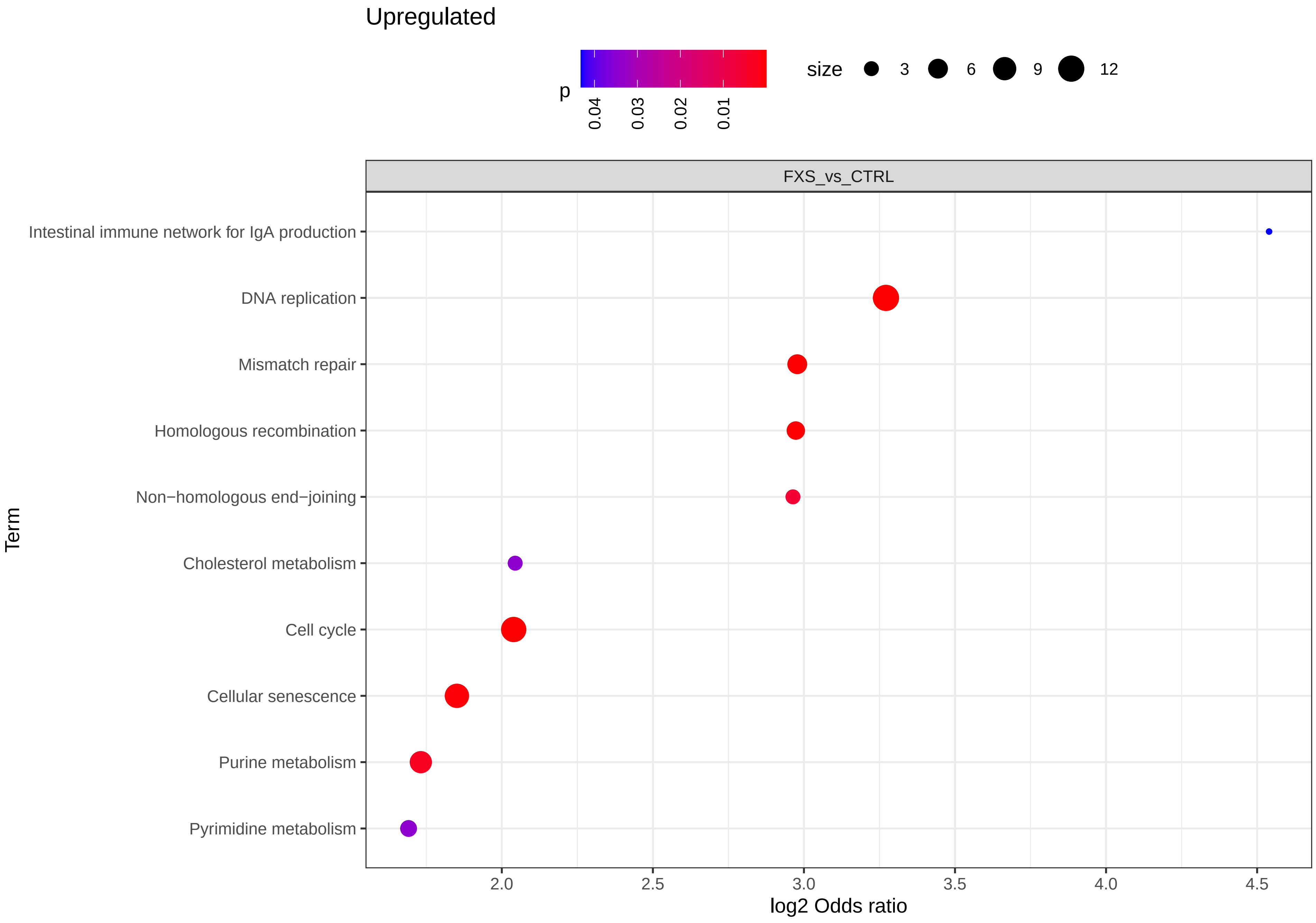

Upregulated
p
0.04
0.03
0.02
0.01
size
3
6
9
12
FXS_vs_CTRL
Intestinal immune network for IgA production
DNA replication
Mismatch repair
Homologous recombination
Non−homologous end−joining
Cholesterol metabolism
Cell cycle
Cellular senescence
Purine metabolism
Pyrimidine metabolism
Term
2.0
2.5
3.0
3.5
4.0
4.5
log2 Odds ratio

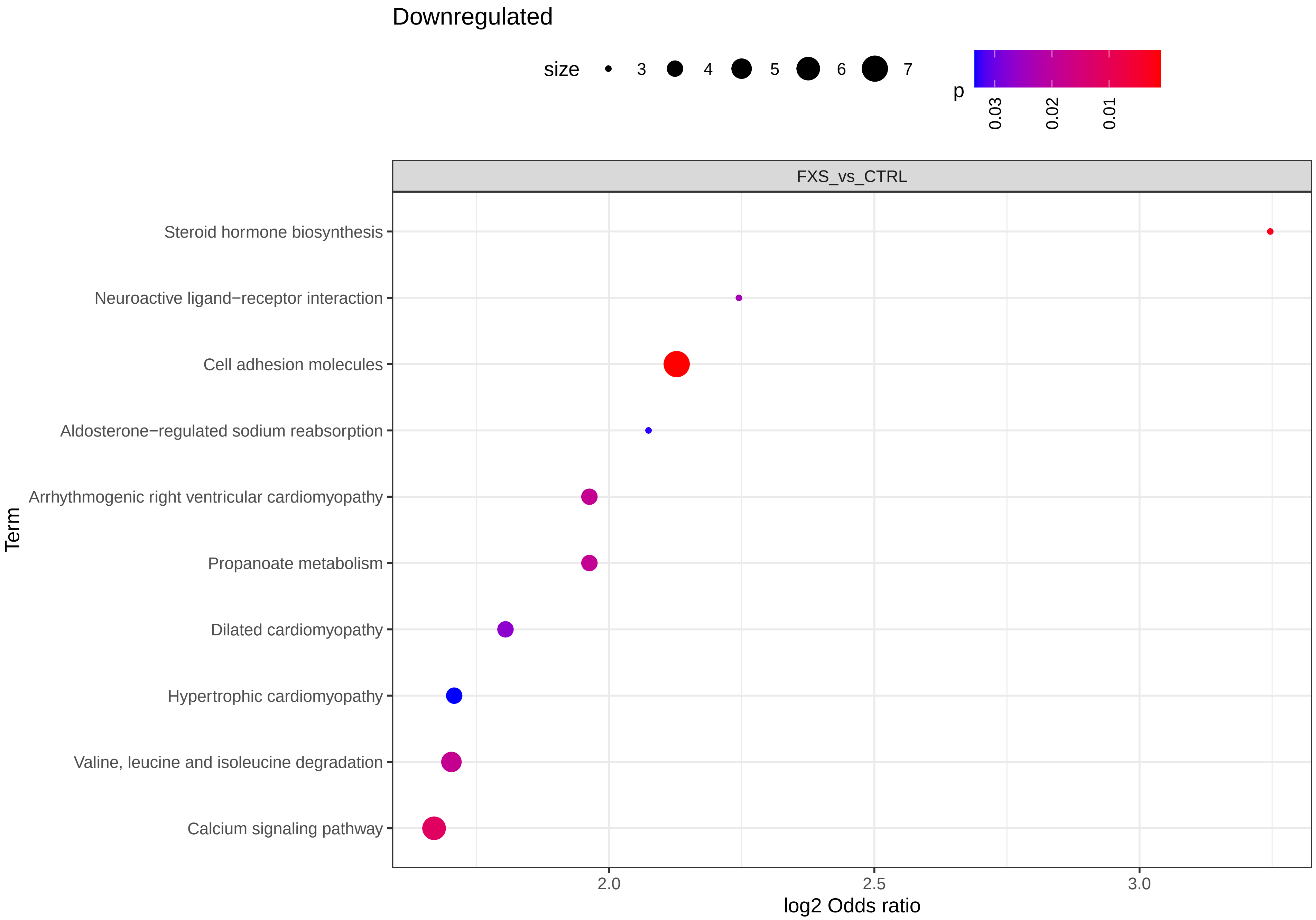

Downregulated
size
3
4
5
6
7
p
0.03
0.02
0.01
FXS_vs_CTRL
Steroid hormone biosynthesis
Neuroactive ligand−receptor interaction
Cell adhesion molecules
Aldosterone−regulated sodium reabsorption
Arrhythmogenic right ventricular cardiomyopathy
Propanoate metabolism
Dilated cardiomyopathy
Hypertrophic cardiomyopathy
Valine, leucine and isoleucine degradation
Calcium signaling pathway
Term
2.0
2.5
3.0
log2 Odds ratio
