# Supplementary material for: Frag’n’Flow: automated workflow for large-scale quantitative proteomics in high performance computing environments
Source: BMC Bioinformatics. 2026 Jan 4;27:18. doi: 10.1186/s12859-025-06305-y (PMC12828970; doi:10.1186/s12859-025-06305-y)
Supplement: Supplementary file 2 — Additional file 2: Data S2. Contains the results and output files from the DIA case study. Table S2. (CSV file) includes the identified protein intensities and differential expression analysis results, based on the data-independent acquisition workflow. Output S2. (PDF file) is the FP-Analyst generated report with all corresponding visualizations and outputs. [file 12859_2025_6305_MOESM2_ESM.zip › Data S2/Output S2.pdf]

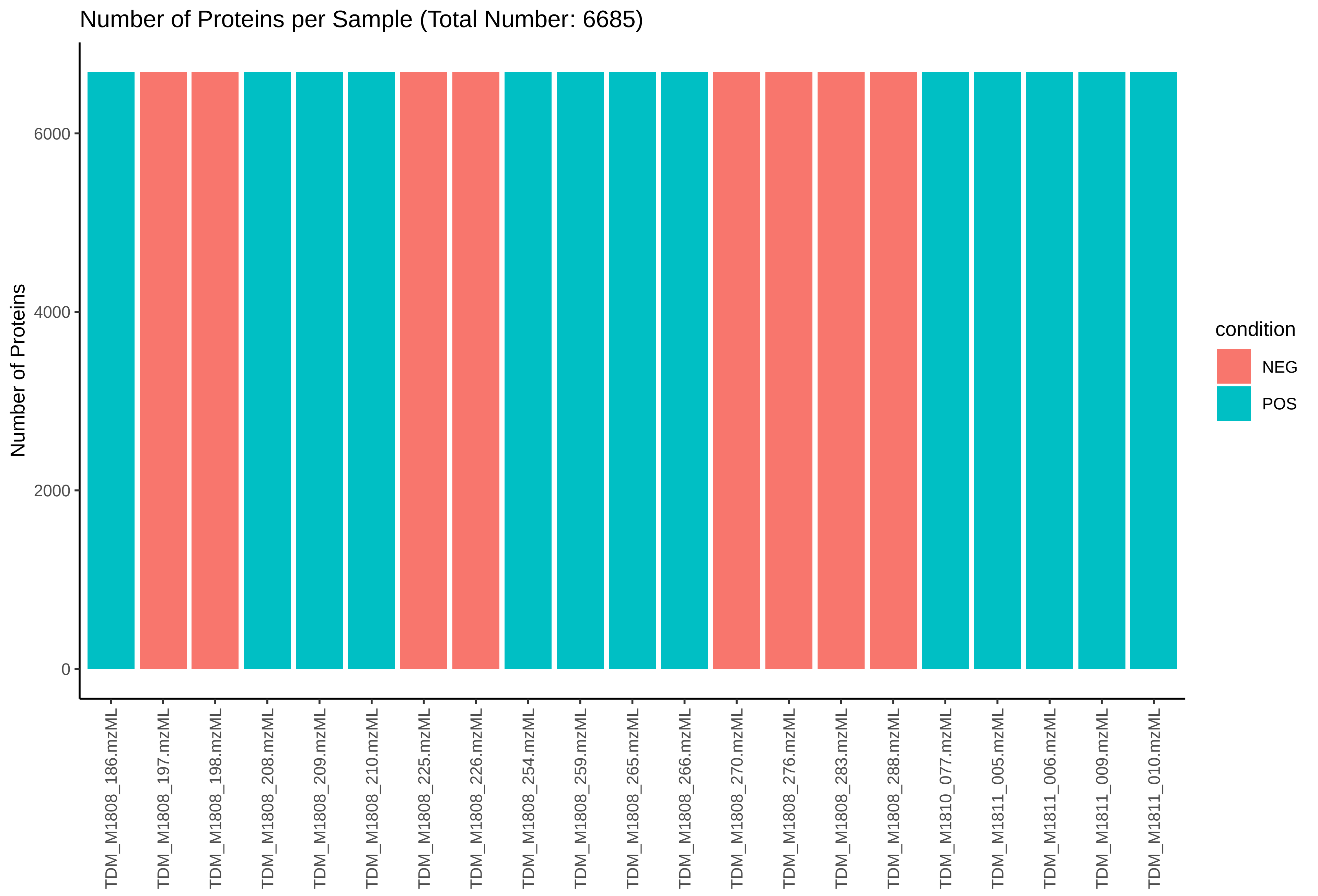

Number of Proteins per Sample (Total Number: 6685)
Number of Proteins
0
2000
4000
6000
condition
NEG
POS
TDM_M1808_186.mzML
TDM_M1808_197.mzML
TDM_M1808_198.mzML
TDM_M1808_208.mzML
TDM_M1808_209.mzML
TDM_M1808_210.mzML
TDM_M1808_225.mzML
TDM_M1808_226.mzML
TDM_M1808_254.mzML
TDM_M1808_259.mzML
TDM_M1808_265.mzML
TDM_M1808_266.mzML
TDM_M1808_270.mzML
TDM_M1808_276.mzML
TDM_M1808_283.mzML
TDM_M1808_288.mzML
TDM_M1810_077.mzML
TDM_M1811_005.mzML
TDM_M1811_006.mzML
TDM_M1811_009.mzML
TDM_M1811_010.mzML

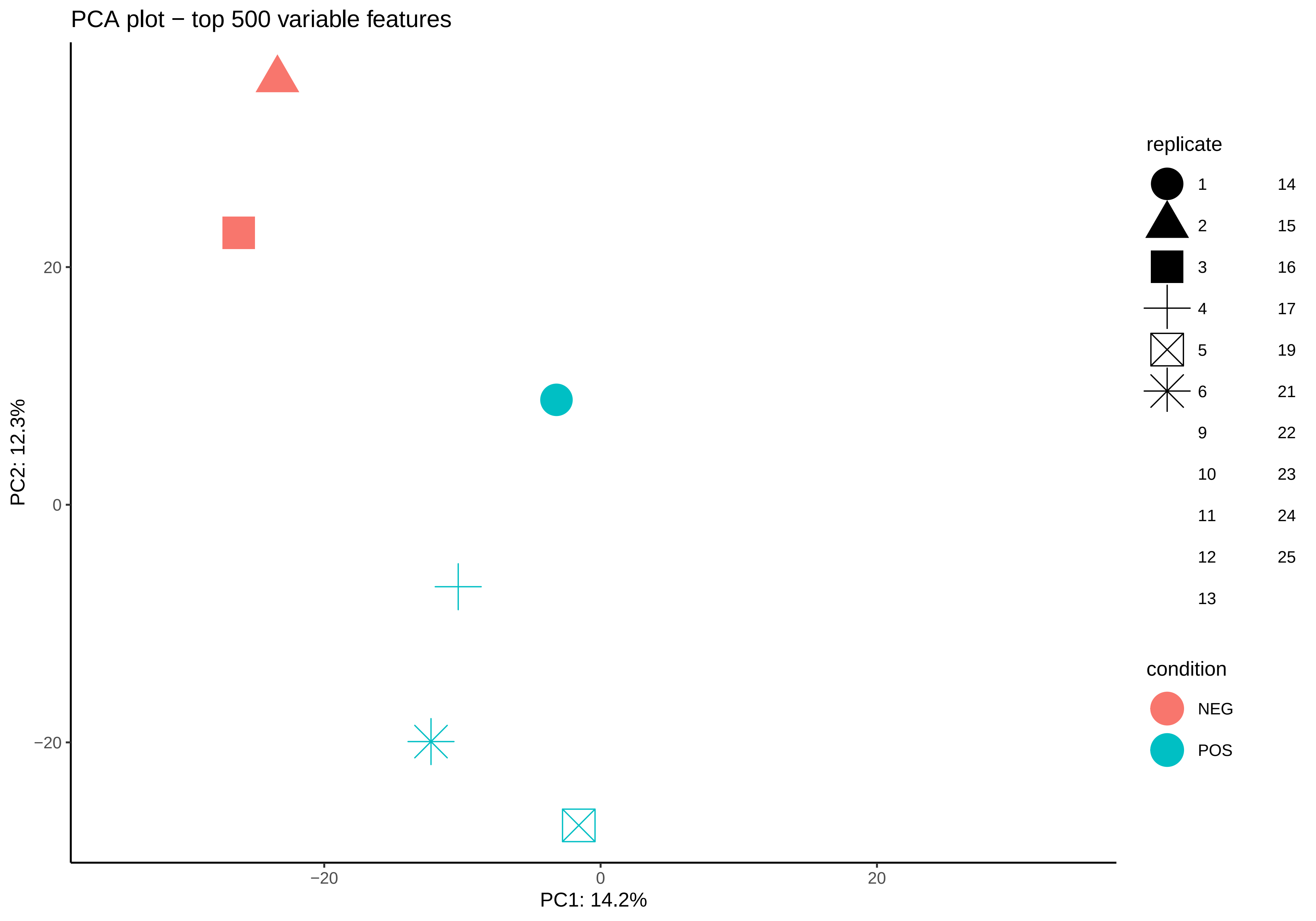
PCA plot − top 500 variable features
PC1: 14.2%
PC2: 12.3%
−20
0
20
20
0
−20
replicate
1
2
3
4
5
6
9
10
11
12
13
14
15
16
17
19
21
22
23
24
25
condition
NEG
POS

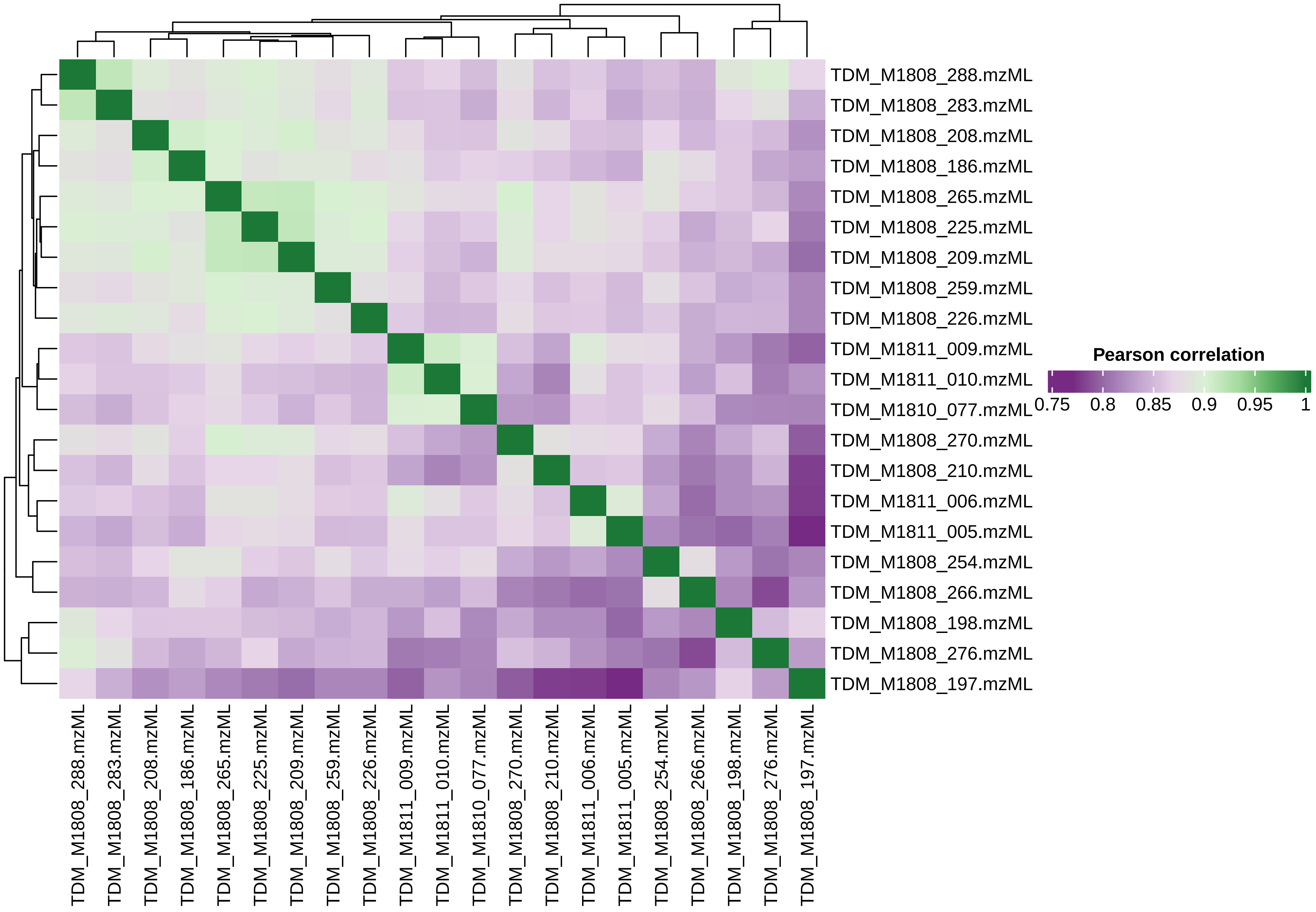

TDM_M1808_288.mzML
TDM_M1808_283.mzML
TDM_M1808_208.mzML
TDM_M1808_186.mzML
TDM_M1808_265.mzML
TDM_M1808_225.mzML
TDM_M1808_209.mzML
TDM_M1808_259.mzML
TDM_M1808_226.mzML
TDM_M1811_009.mzML
TDM_M1811_010.mzML
TDM_M1810_077.mzML
TDM_M1808_270.mzML
TDM_M1808_210.mzML
TDM_M1811_006.mzML
TDM_M1811_005.mzML
TDM_M1808_254.mzML
TDM_M1808_266.mzML
TDM_M1808_198.mzML
TDM_M1808_276.mzML
TDM_M1808_197.mzML
Pearson correlation
0.75
0.8
0.85
0.9
0.95
1

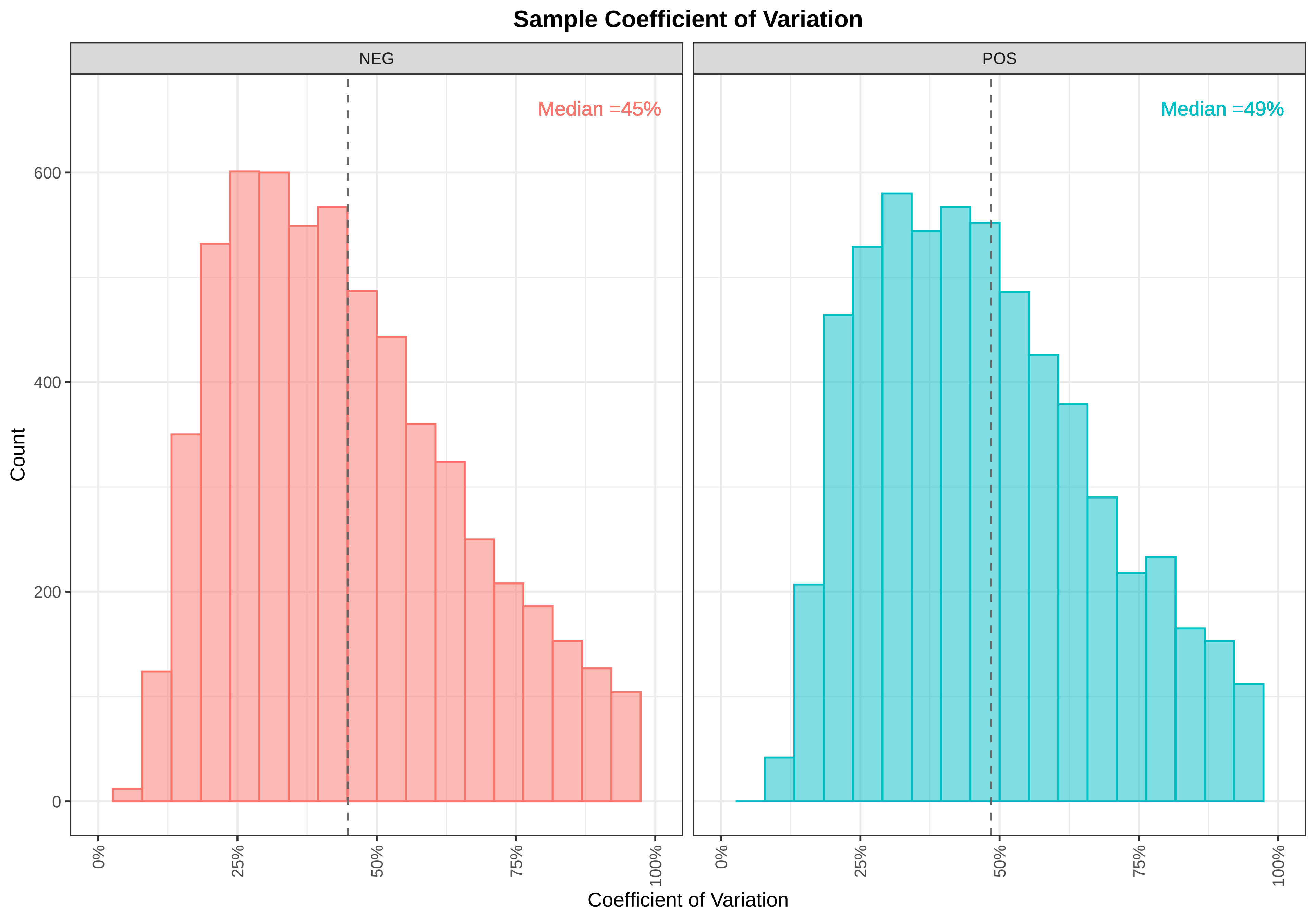

Sample Coefficient of Variation
NEG
POS
Median =45%
Median =49%
Count
0
200
400
600
0%
25%
50%
75%
100%
Coefficient of Variation

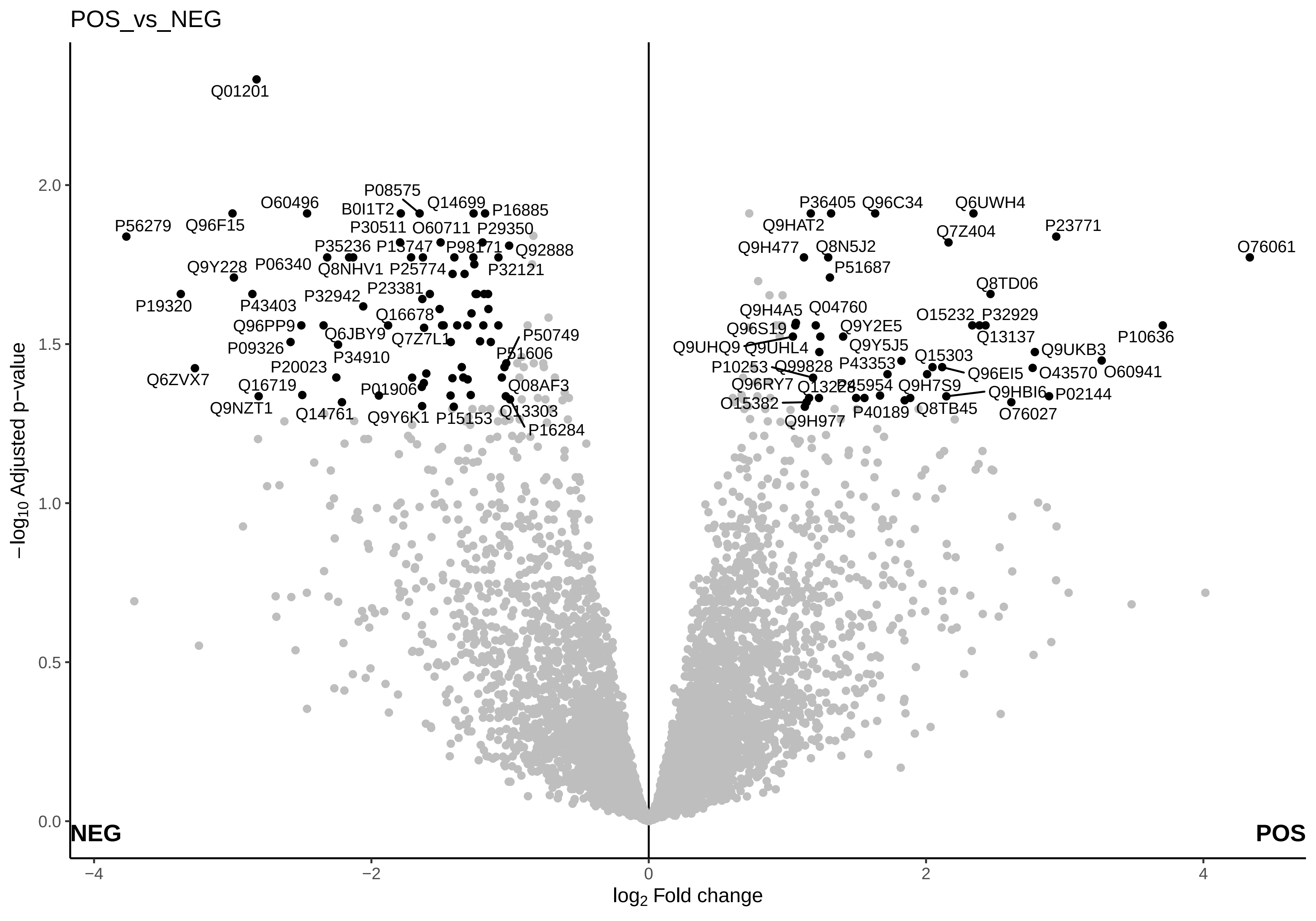

POS_vs_NEG
NEG
POS
−log10 Adjusted p−value
log2 Fold change
0.0
0.5
1.0
1.5
2.0
−4
−2
0
2
4
Q01201
P56279
Q96F15
O60496
P08575
B0I1T2
Q14699
P16885
P30511
O60711
P29350
P35236
Q92888
Q8NHV1
P25774
P32121
Q9Y228
P06340
P19320
P43403
P32942
P23381
Q16678
Q96PP9
Q6JBY9
Q7Z7L1
P50749
P09326
P51606
P34910
P20023
Q6ZVX7
Q16719
P01906
Q08AF3
Q9NZT1
Q14761
Q9Y6K1
P15153
Q13303
P16284
P36405
Q96C34
Q6UWH4
Q9HAT2
Q7Z404
P23771
O76061
Q9H477
Q8N5J2
P51687
Q8TD06
Q9H4A5
Q04760
O15232
P32929
Q96S19
Q9Y2E5
Q13137
P10636
Q9UHQ9
Q9UHL4
Q9Y5J5
Q9UKB3
P10253
Q99828
P43353
Q15303
Q96EI5
O43570
O60941
Q96RY7
Q9H7S9
Q9HBI6
P02144
O15382
P40189
Q8TB45
O76027
Q9H977

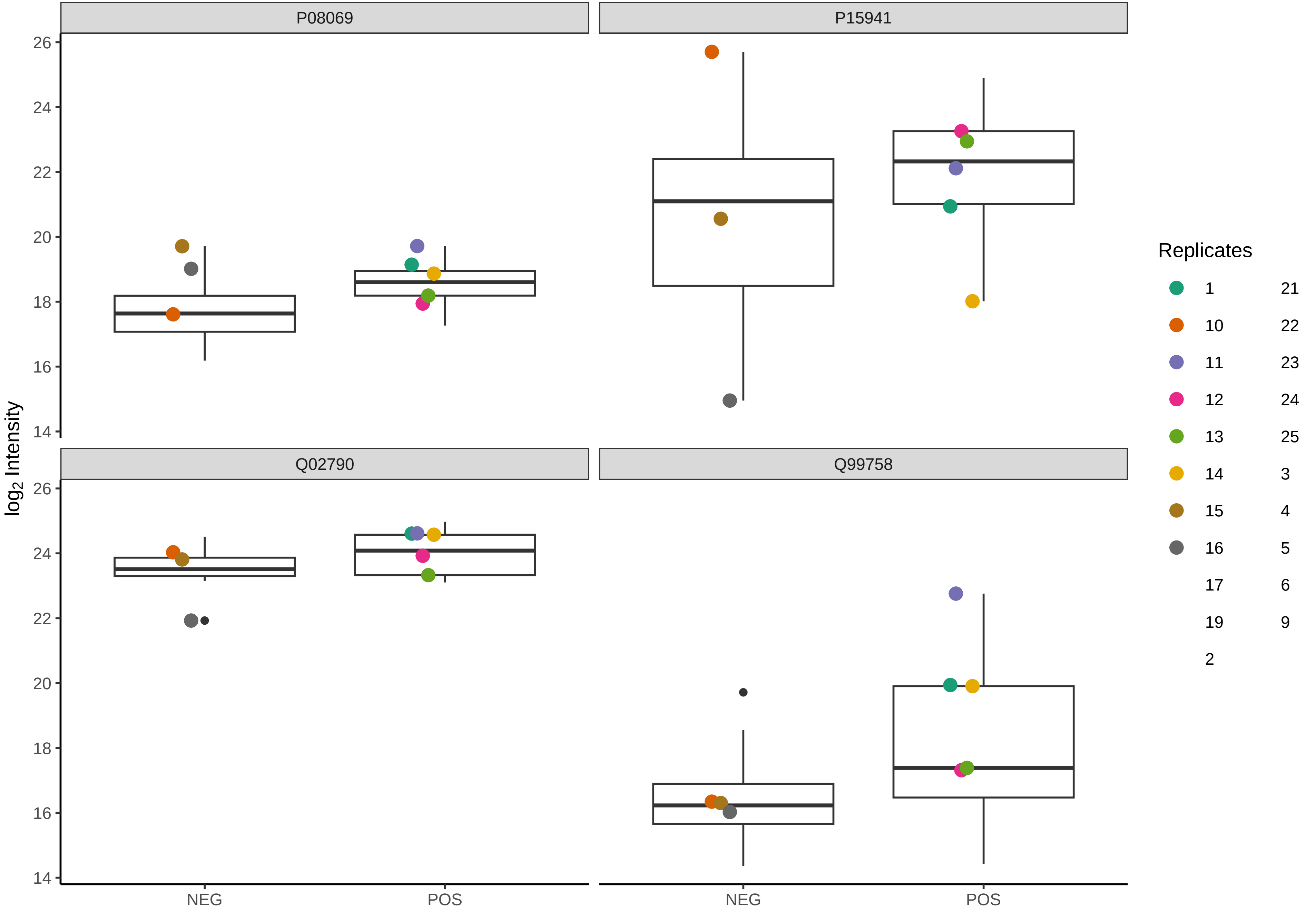
P08069
P15941
Q02790
Q99758
log2 Intensity
26
24
22
20
18
16
14
NEG
POS
Replicates
1
10
11
12
13
14
15
16
17
19
2
21
22
23
24
25
3
4
5
6
9

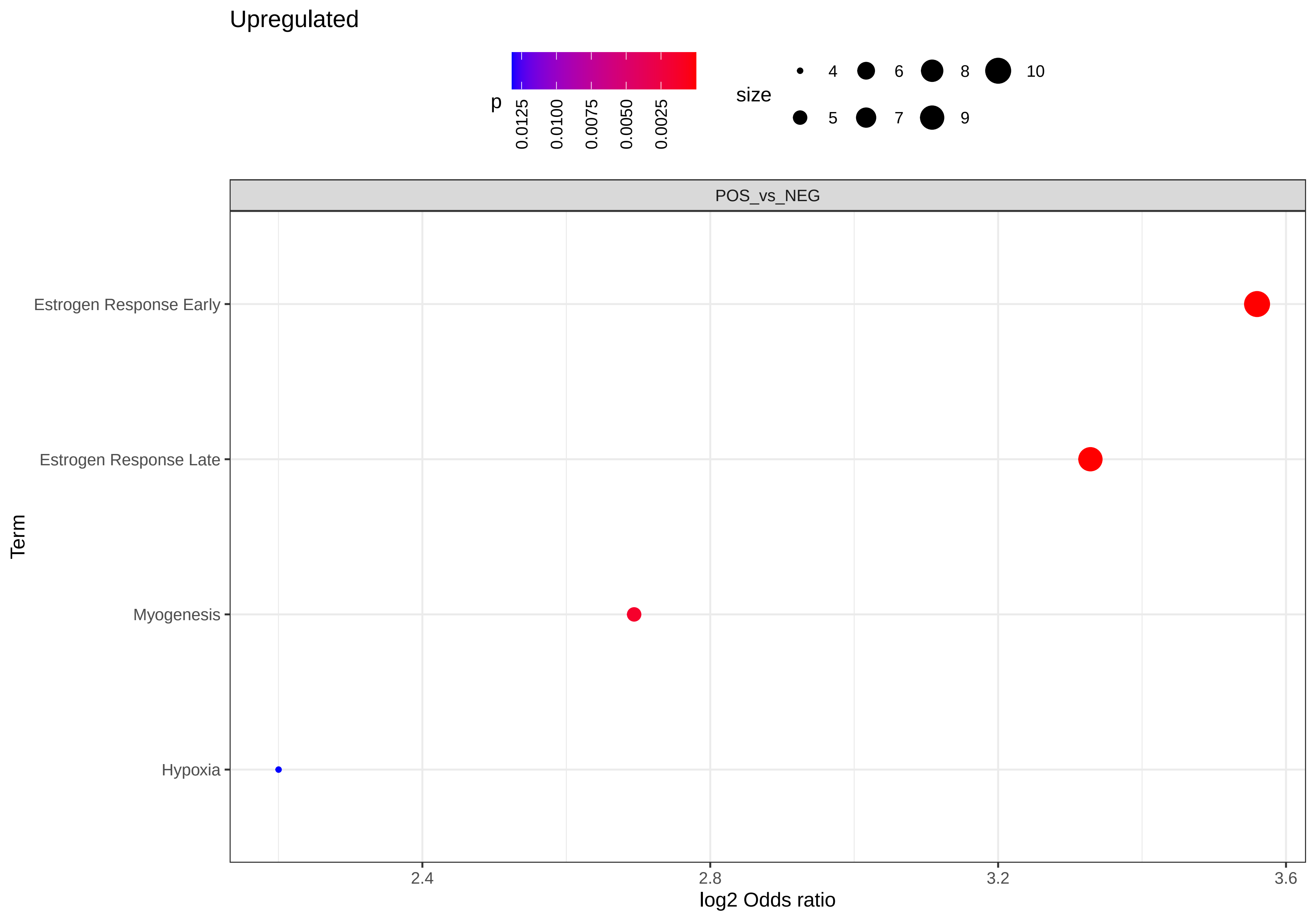

Upregulated
p
0.0125
0.0100
0.0075
0.0050
0.0025
size
4
5
6
7
8
9
10
POS_vs_NEG
Estrogen Response Early
Estrogen Response Late
Myogenesis
Hypoxia
Term
2.4
2.8
3.2
3.6
log2 Odds ratio

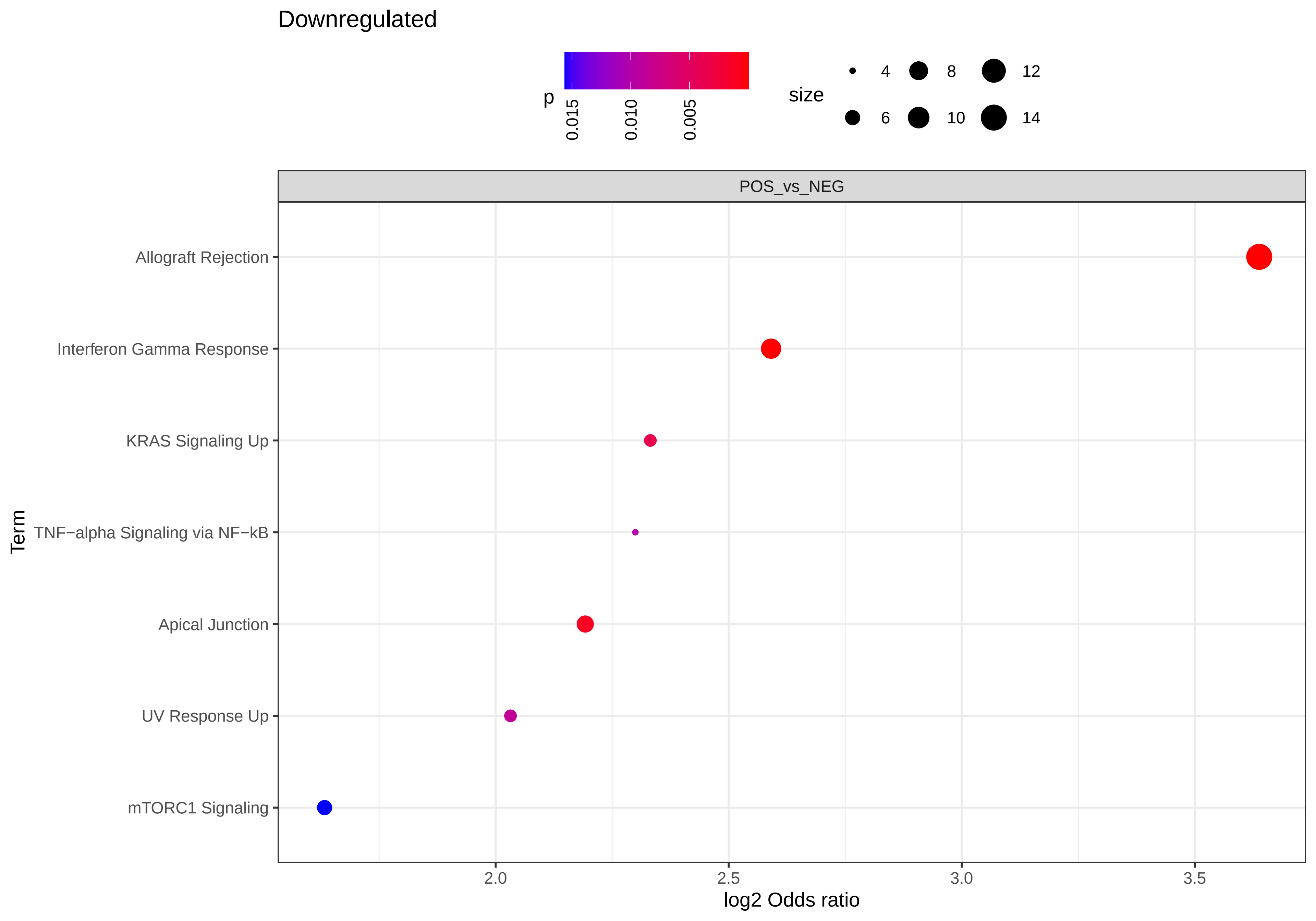

Downregulated
p
0.015
0.010
0.005
size
4
8
12
6
10
14
POS_vs_NEG
Allograft Rejection
Interferon Gamma Response
KRAS Signaling Up
TNF−alpha Signaling via NF−kB
Apical Junction
UV Response Up
mTORC1 Signaling
Term
2.0
2.5
3.0
3.5
log2 Odds ratio
