# Supplementary material for: Frag’n’Flow: automated workflow for large-scale quantitative proteomics in high performance computing environments
Source: BMC Bioinformatics. 2026 Jan 4;27:18. doi: 10.1186/s12859-025-06305-y (PMC12828970; doi:10.1186/s12859-025-06305-y)
Supplement: Supplementary file 3 — Additional file 3. Data S3. Contains the results and output files from the TMT case study. Table S3. (CSV file) provides quantified protein intensities and differential expression analysis results obtained from the tandem mass tag (TMT) experiments. Output S3. (PDF file) is the FP-Analyst generated report with all corresponding visualizations and outputs. [file 12859_2025_6305_MOESM3_ESM.zip › Data S3/Output S3.pdf]

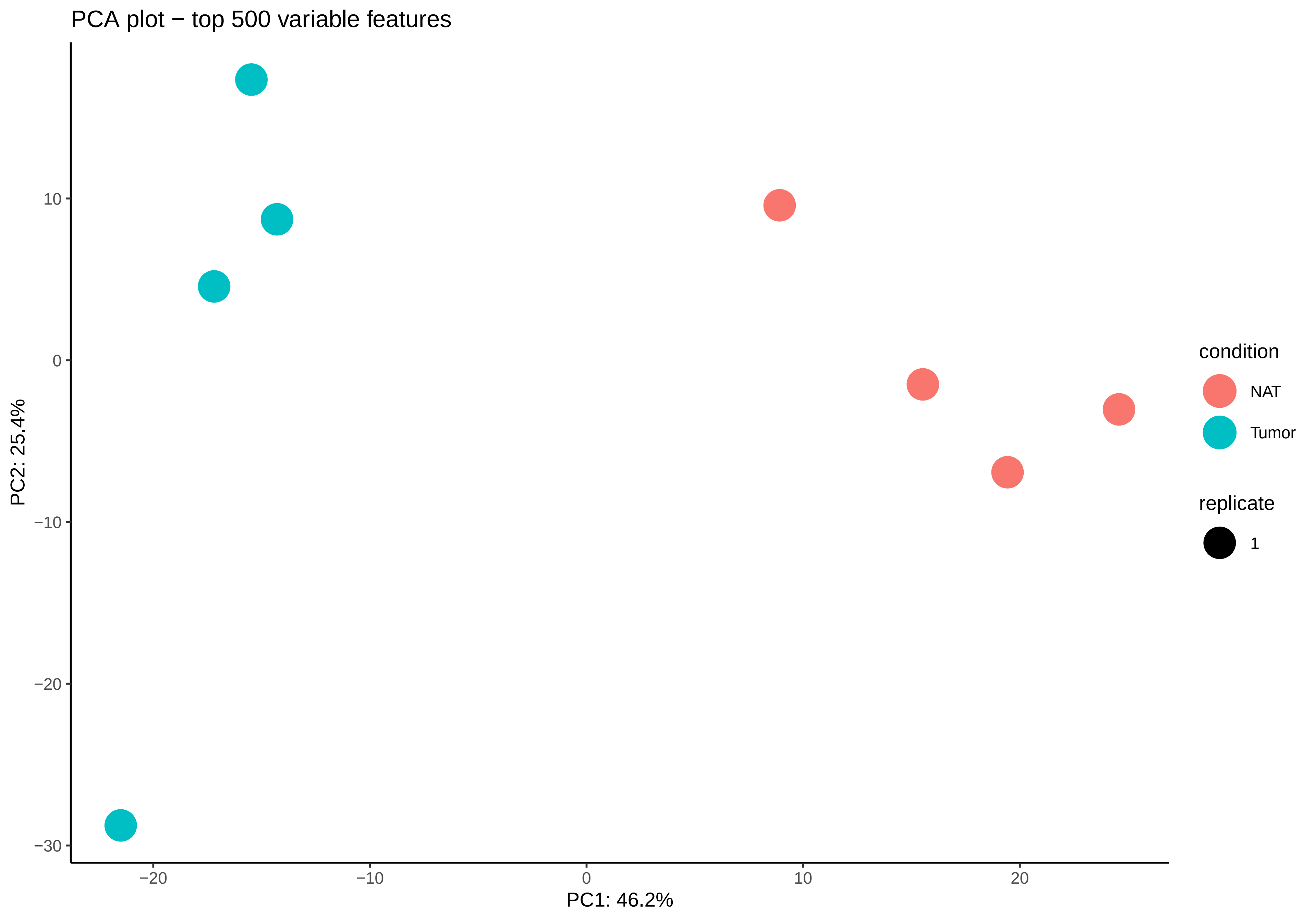
PCA plot − top 500 variable features
PC2: 25.4%
PC1: 46.2%
10
0
−10
−20
−30
−20
−10
0
10
20
condition
NAT
Tumor
replicate
1

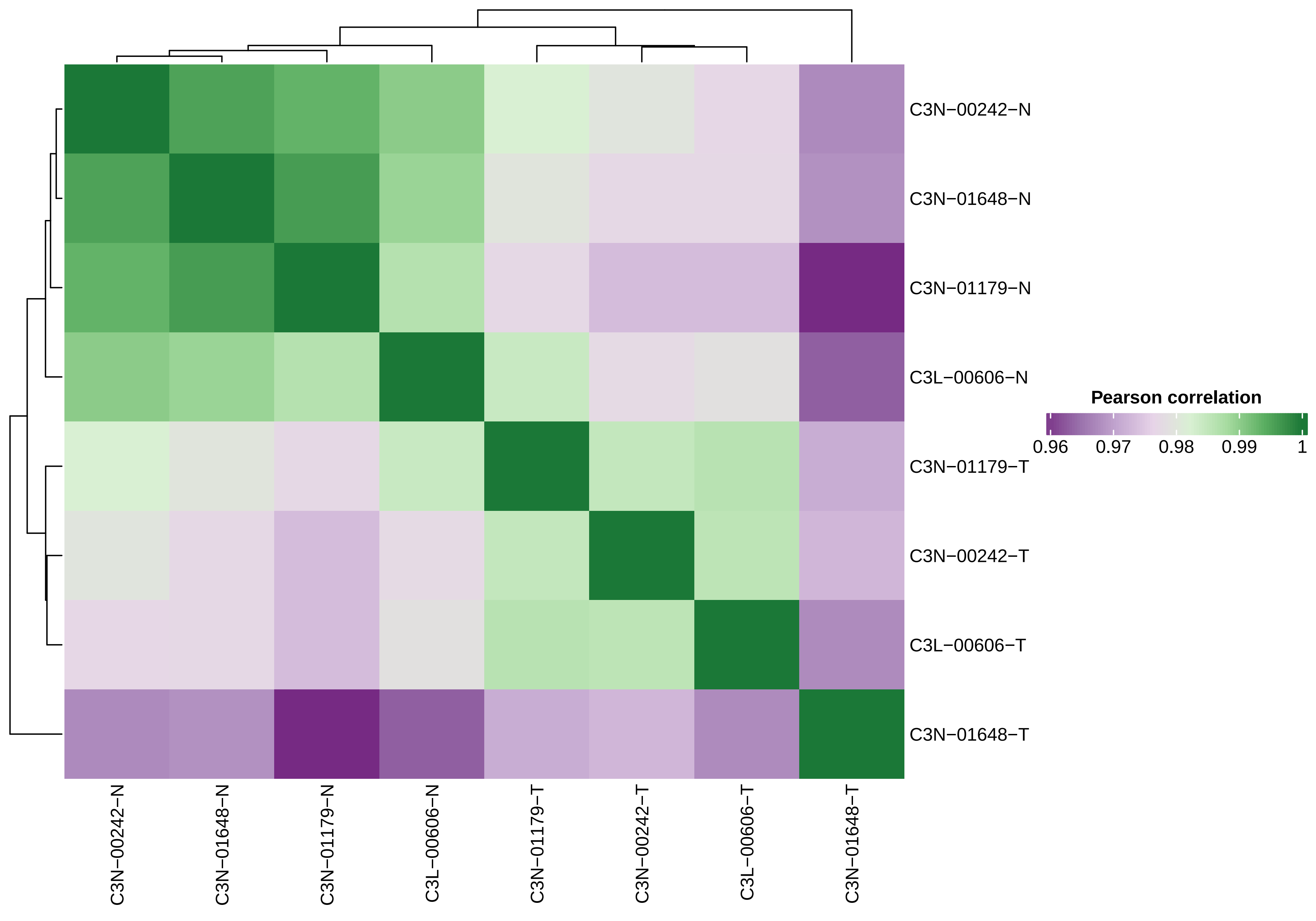
C3N−00242−N
C3N−01648−N
C3N−01179−N
C3L−00606−N
C3N−01179−T
C3N−00242−T
C3L−00606−T
C3N−01648−T
Pearson correlation
0.96
0.97
0.98
0.99
1

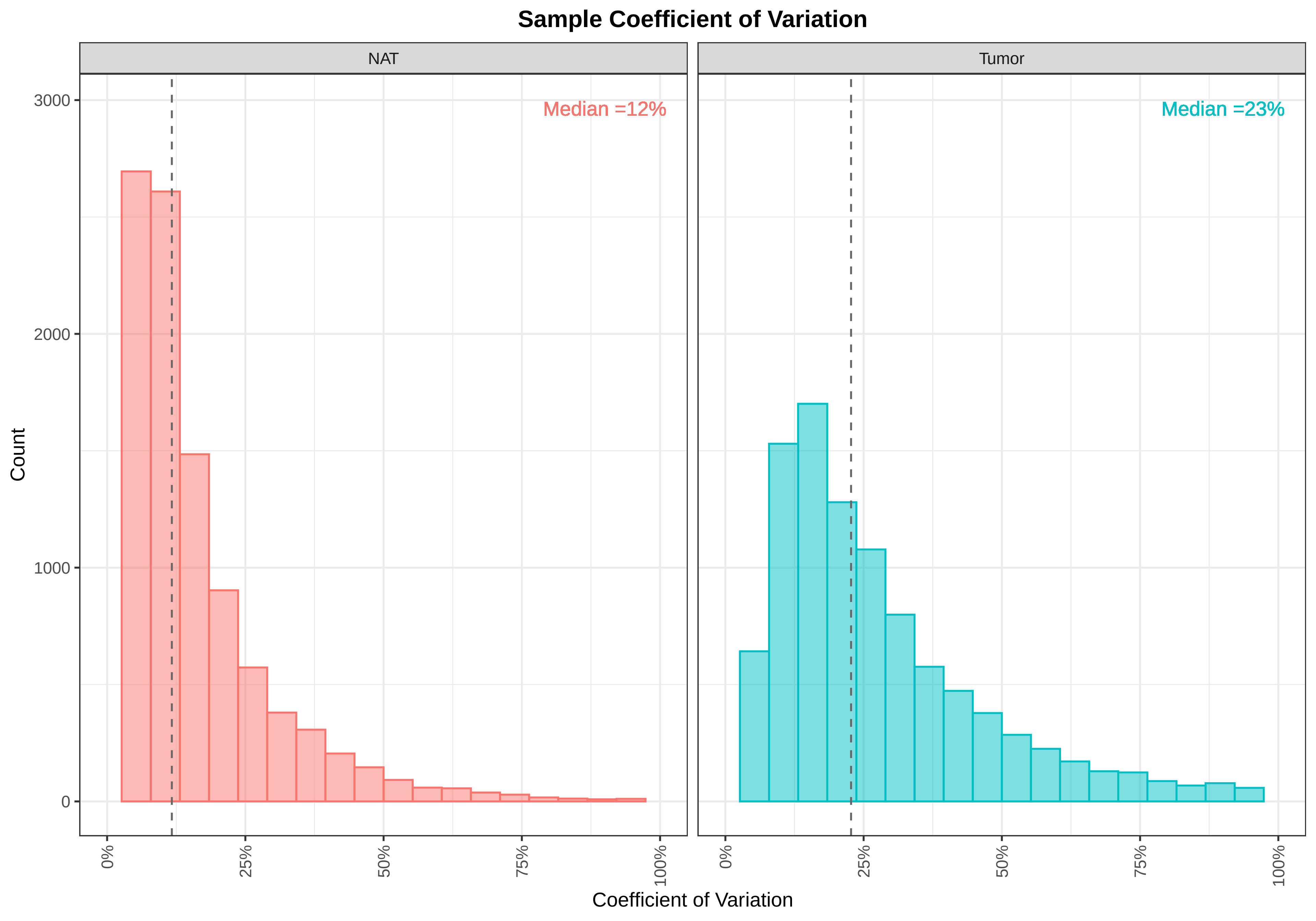

Sample Coefficient of Variation
NAT
Tumor
Median =12%
Median =23%
Count
0
1000
2000
3000
0%
25%
50%
75%
100%
Coefficient of Variation

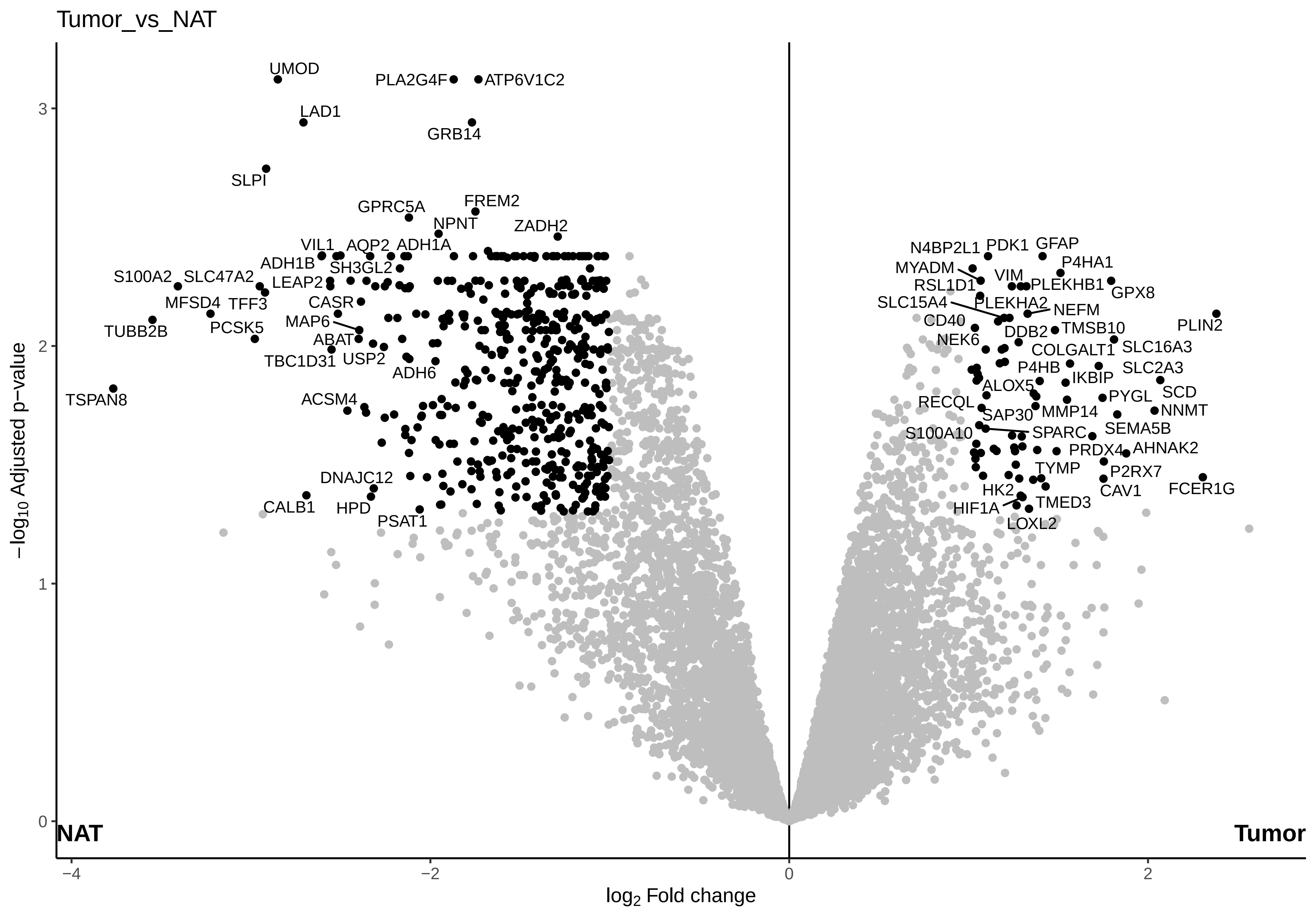

Tumor_vs_NAT
UMOD
PLA2G4F
ATP6V1C2
LAD1
GRB14
SLPI
GPRC5A
FREM2
NPNT
ZADH2
VIL1
AQP2
ADH1A
ADH1B
SH3GL2
S100A2
SLC47A2
LEAP2
MFSD4
TFF3
CASR
MAP6
TUBB2B
PCSK5
ABAT
TBC1D31
USP2
ADH6
TSPAN8
ACSM4
DNAJC12
CALB1
HPD
PSAT1
N4BP2L1
PDK1
GFAP
MYADM
P4HA1
VIM
RSL1D1
PLEKHB1
GPX8
SLC15A4
PLEKHA2
NEFM
CD40
PLIN2
DDB2
TMSB10
NEK6
SLC16A3
COLGALT1
P4HB
SLC2A3
IKBIP
ALOX5
SCD
RECQL
PYGL
SAP30
MMP14
NNMT
S100A10
SPARC
SEMA5B
PRDX4
AHNAK2
TYMP
P2RX7
HK2
CAV1
FCER1G
HIF1A
TMED3
LOXL2
NAT
Tumor
3
2
1
0
−4
−2
0
2
−log10 Adjusted p−value
log2 Fold change

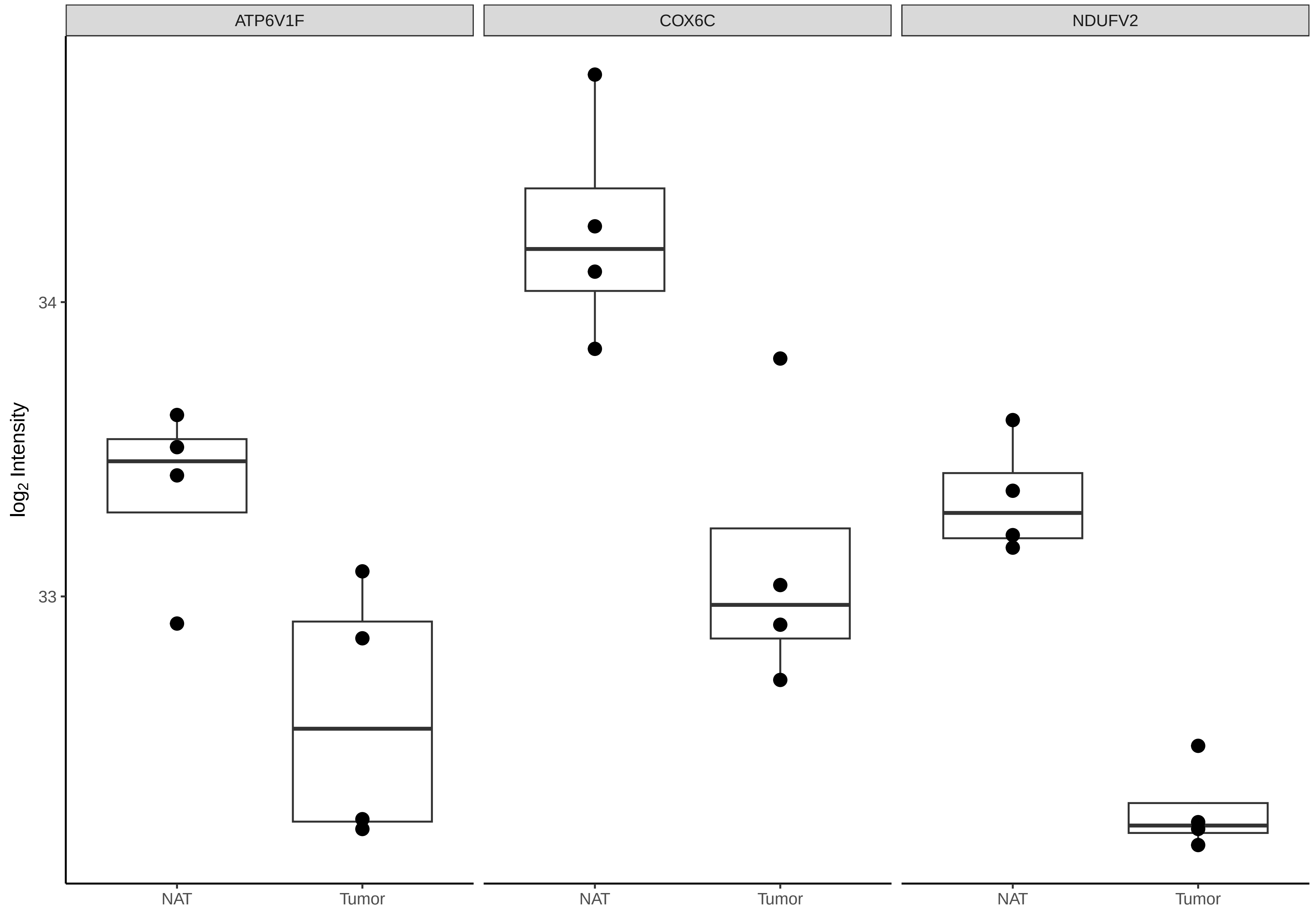
ATP6V1F
COX6C
NDUFV2
log2 Intensity
34
33
NAT
Tumor
NAT
Tumor
NAT
Tumor

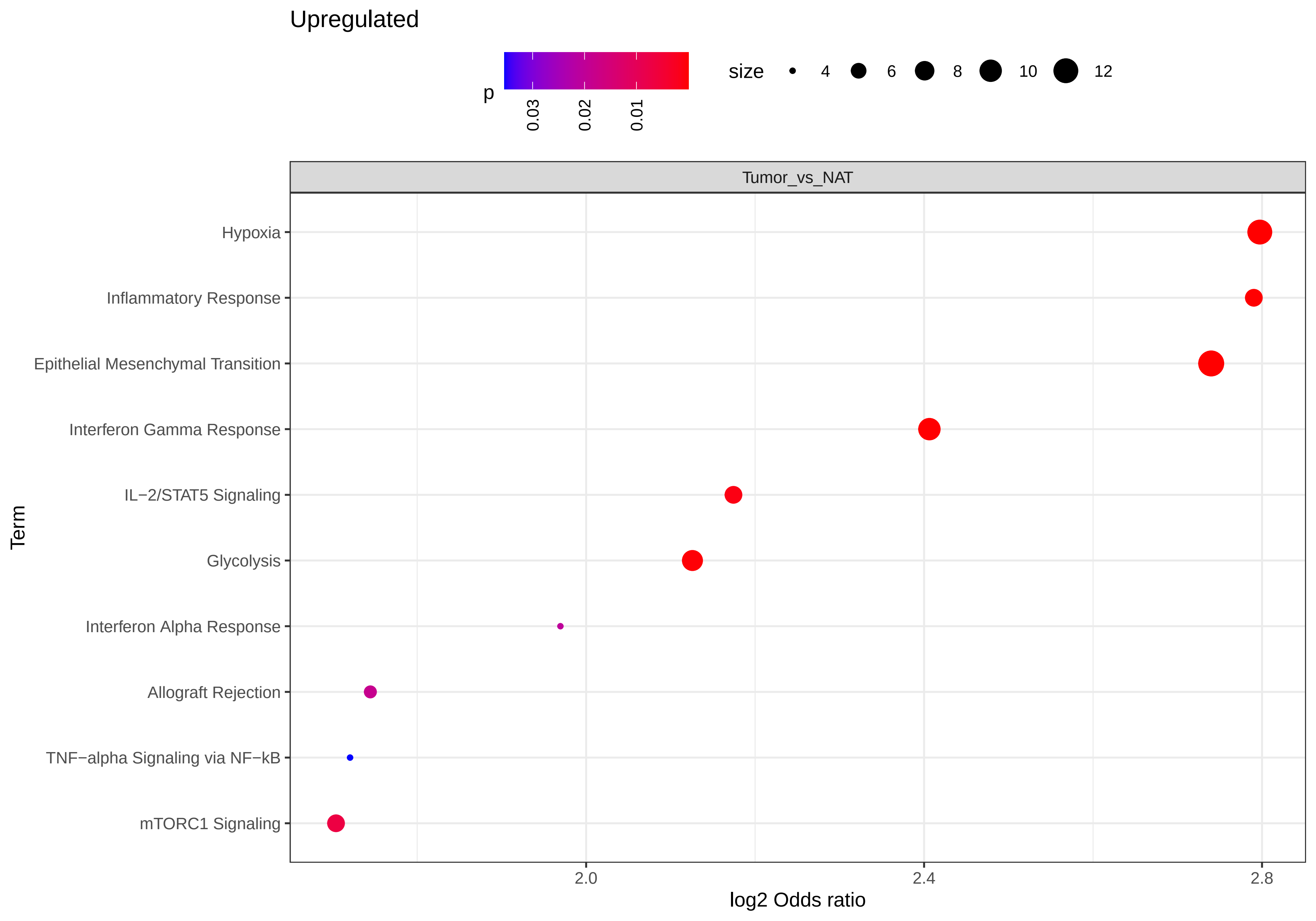

Upregulated
p
0.03
0.02
0.01
size
4
6
8
10
12
Tumor_vs_NAT
Hypoxia
Inflammatory Response
Epithelial Mesenchymal Transition
Interferon Gamma Response
IL−2/STAT5 Signaling
Glycolysis
Interferon Alpha Response
Allograft Rejection
TNF−alpha Signaling via NF−kB
mTORC1 Signaling
Term
2.0
2.4
2.8
log2 Odds ratio

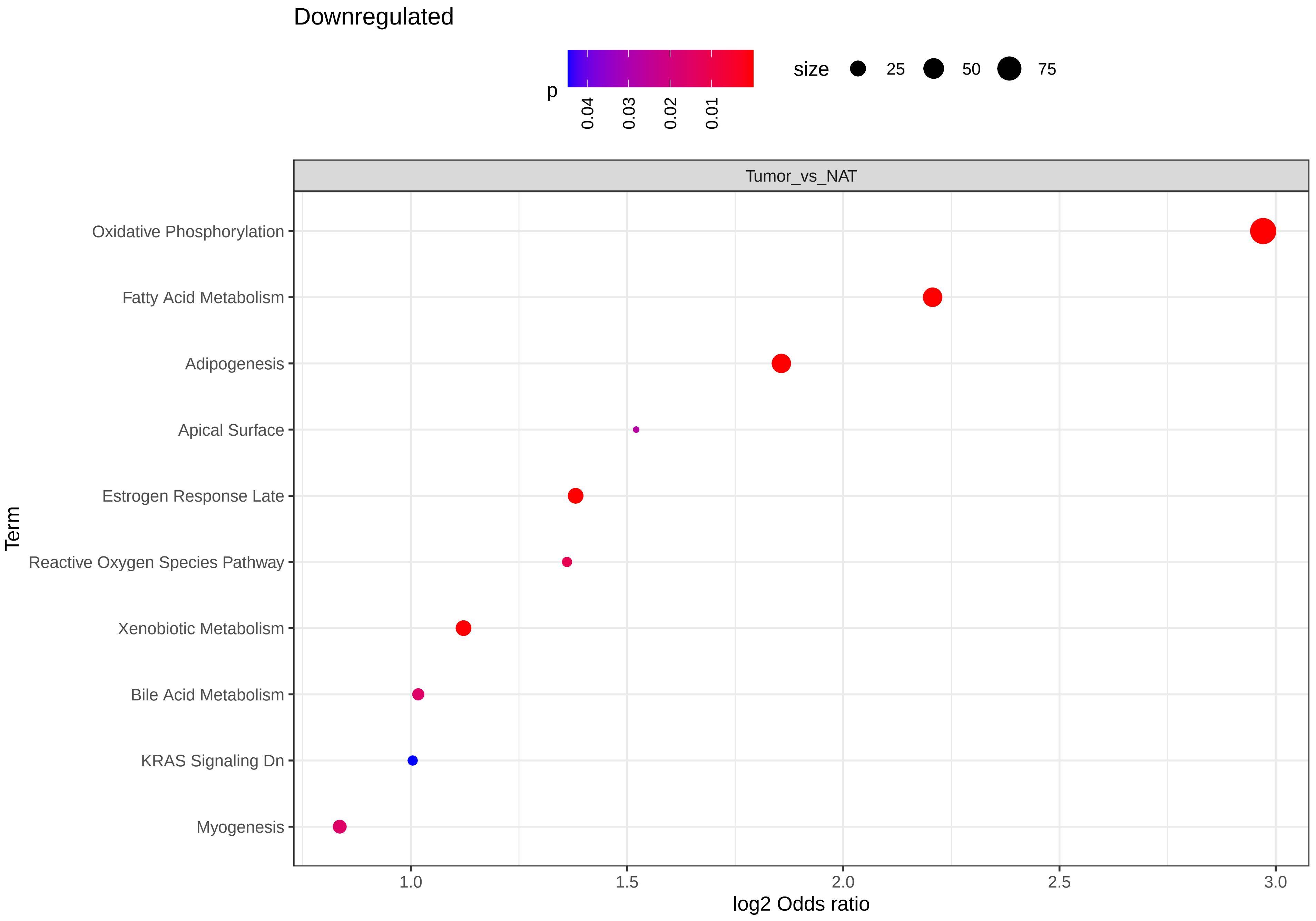

Downregulated
p
0.04
0.03
0.02
0.01
size
25
50
75
Tumor_vs_NAT
Oxidative Phosphorylation
Fatty Acid Metabolism
Adipogenesis
Apical Surface
Estrogen Response Late
Reactive Oxygen Species Pathway
Xenobiotic Metabolism
Bile Acid Metabolism
KRAS Signaling Dn
Myogenesis
Term
1.0
1.5
2.0
2.5
3.0
log2 Odds ratio
