# Supplementary figures and images for: Frag’n’Flow: automated workflow for large-scale quantitative proteomics in high performance computing environments
Source: BMC Bioinformatics. 2026 Jan 4;27:18. doi: 10.1186/s12859-025-06305-y (PMC12828970; doi:10.1186/s12859-025-06305-y)

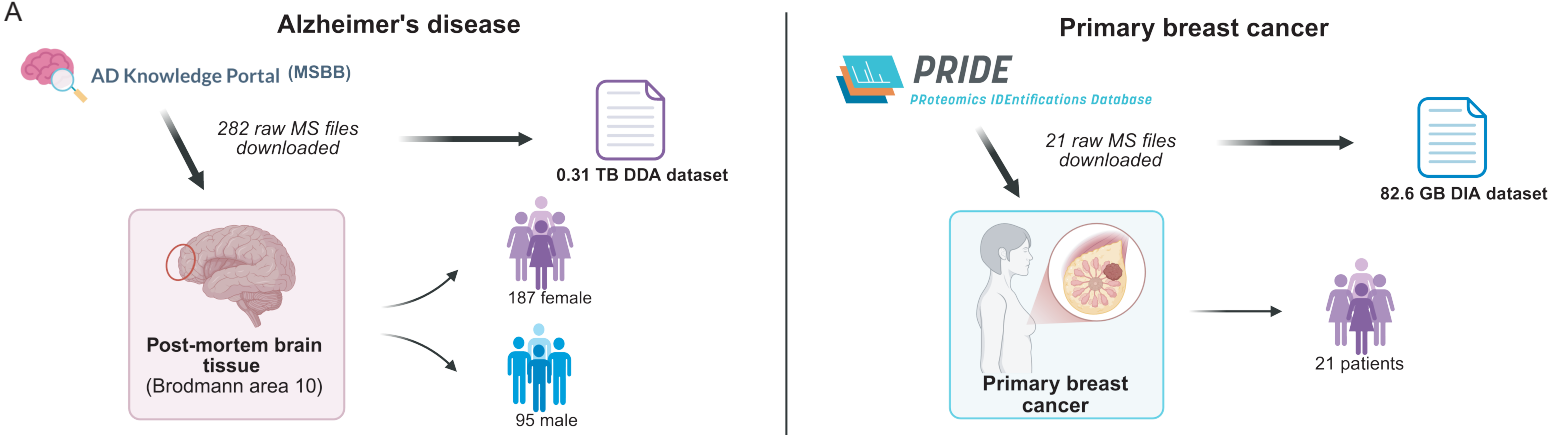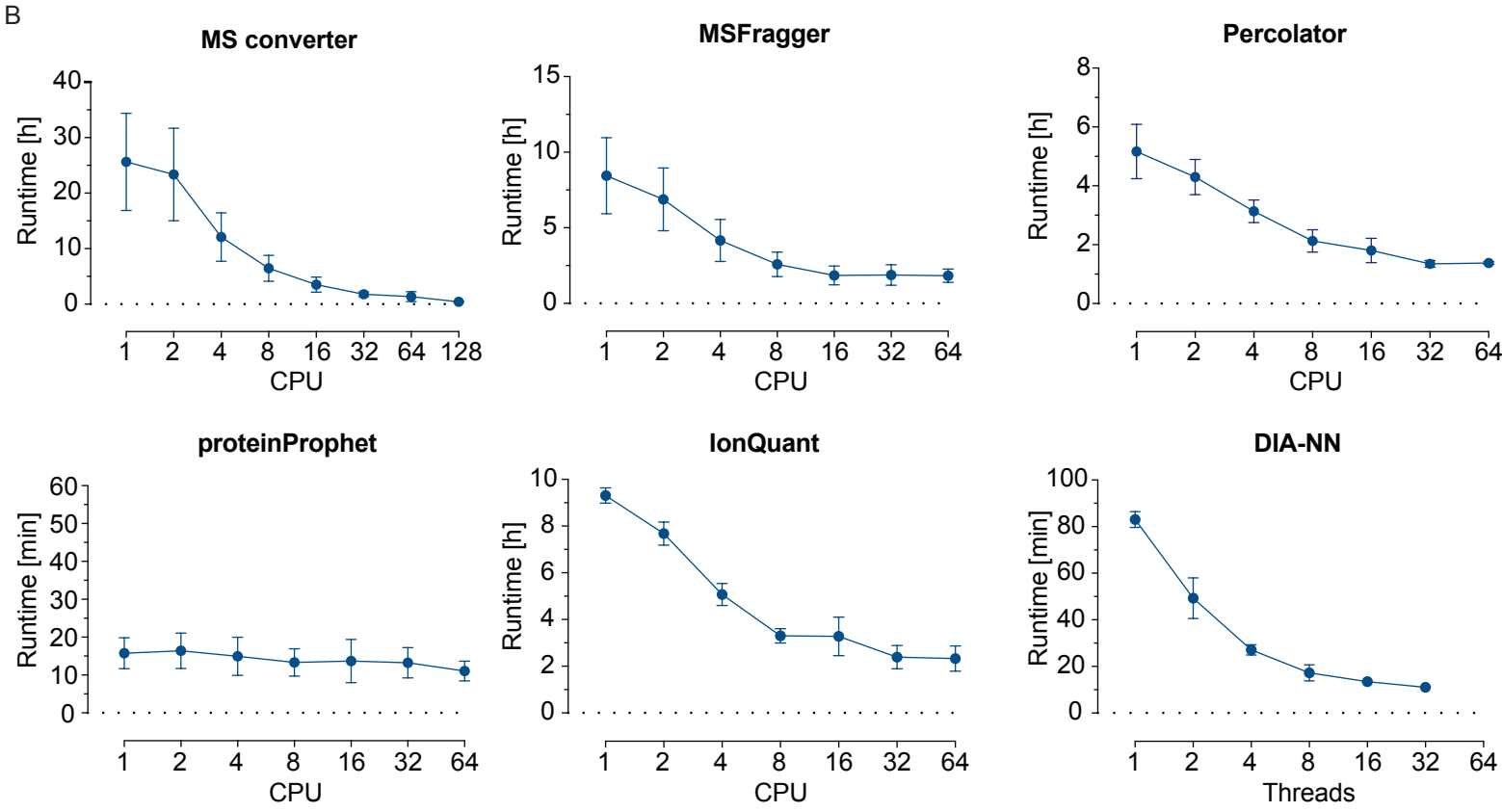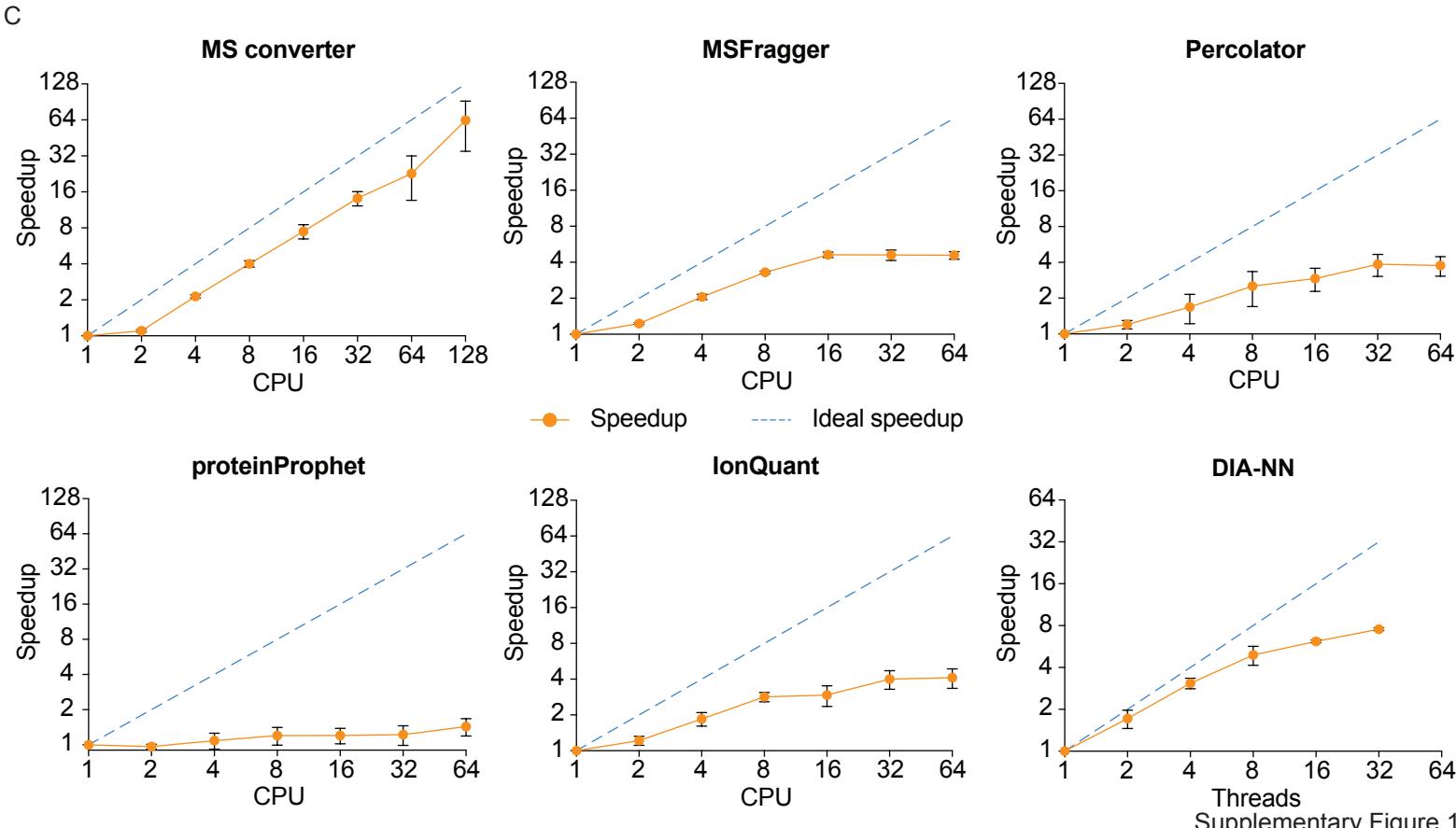

Supplement: Supplementary file 4 — Additional file 4: Figure S1. Benchmark measurements of the individual tools of FragPipe. A Schematics show the experimental design of the case studies used for the benchmarks. B Runtime analysis of individual FP tools with different CPU settings. Error bars indicate standard deviation from 3 independent runs. C Speedup values calculated from the runtime analysis in (B). Error bars indicate standard deviation from 3 independent runs. The dashed line shows the overall speedup trend, while the orange curves represent speedup plateaus corresponding to the local minima observed in (B). [file 12859_2025_6305_MOESM4_ESM.pdf]

A

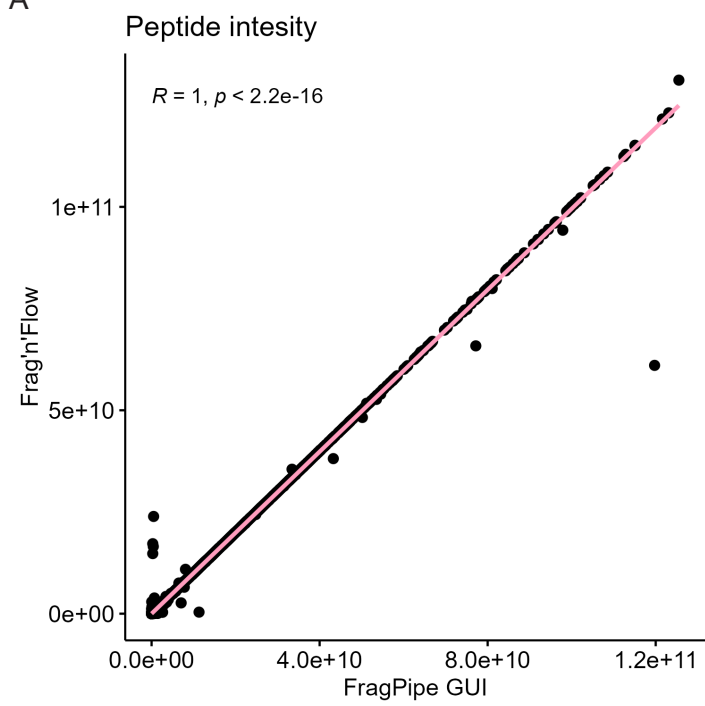

B

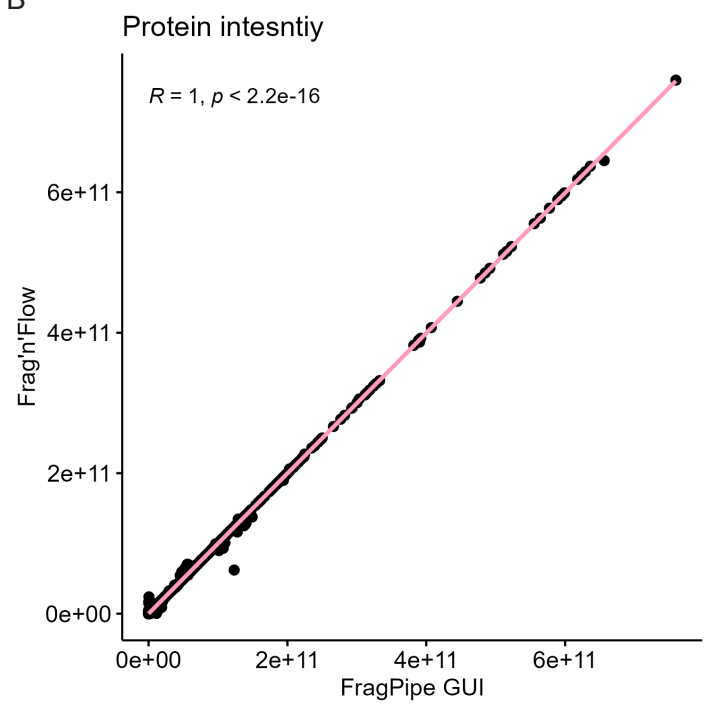

Supplementary Figure 2

Supplement: Supplementary file 5 — Additional file 5: Figure S2. Correlation of peptide (left) and protein (right) intensities between FragPipe analysis methods. Scatter plots comparing peptide and protein intensities obtained from the FragPipe GUI and the Frag’n’Flow pipeline. Peptides and proteins with zero intensity were excluded. Trendlines and Pearson correlation coefficients (R-values) are shown to illustrate the degree of agreement between the two methods. [file 12859_2025_6305_MOESM5_ESM.pdf]

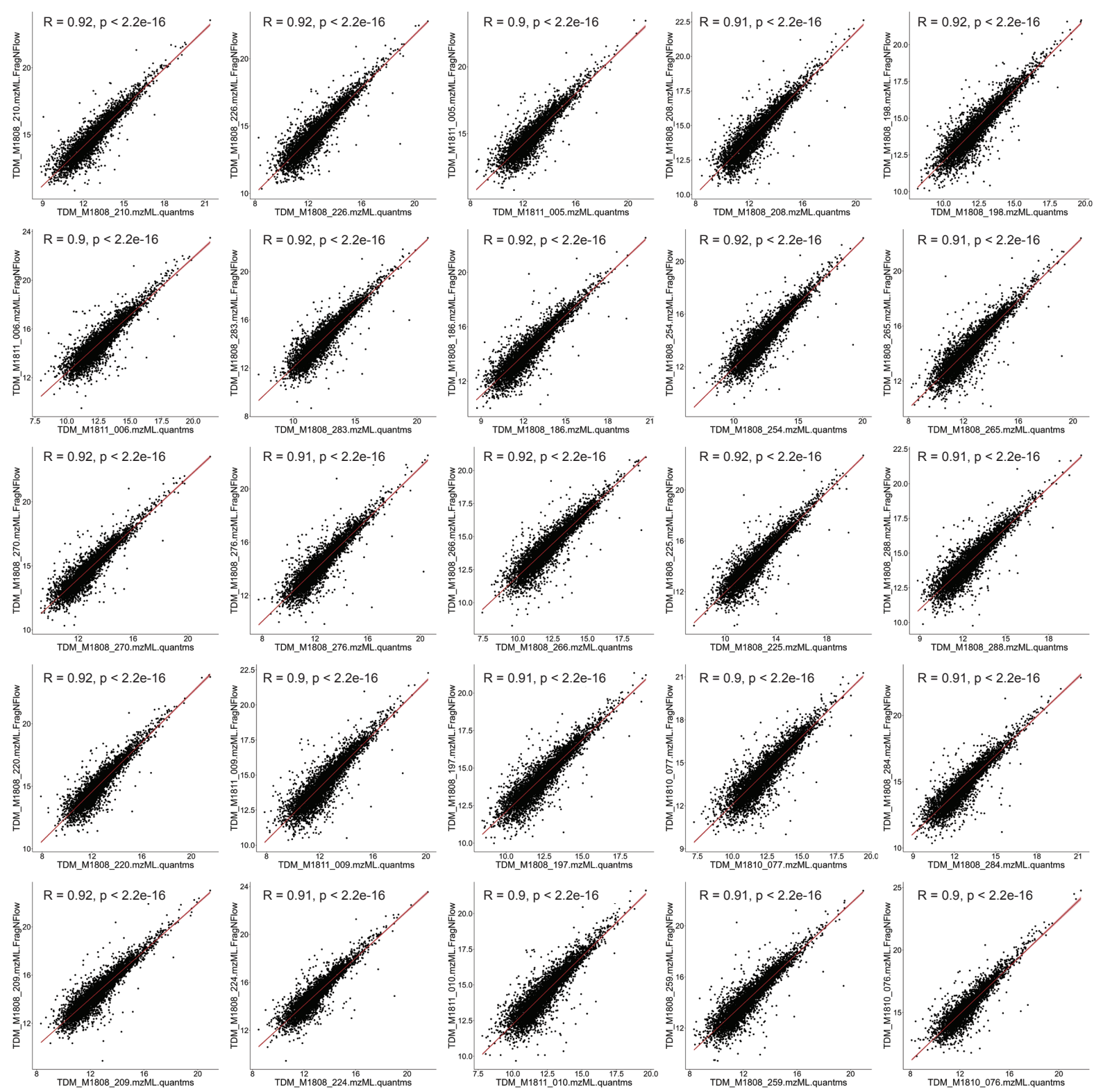

Supplementary Figure 3

Supplement: Supplementary file 6 — Additional file 6: Figure S3. Correlation of normalized protein intensities between samples. Scatter plots comparing log-normalized protein intensities from 25 DIA samples, analysed using quantms and Frag’n’Flow workflows. Each point represents a protein intensity measurement detected in both workflows. Only proteins identified and quantified in both pipelines were plotted. Trendlines and Pearson correlation coefficients (R-values) are shown to illustrate the degree of agreement between the two methods. [file 12859_2025_6305_MOESM6_ESM.pdf]

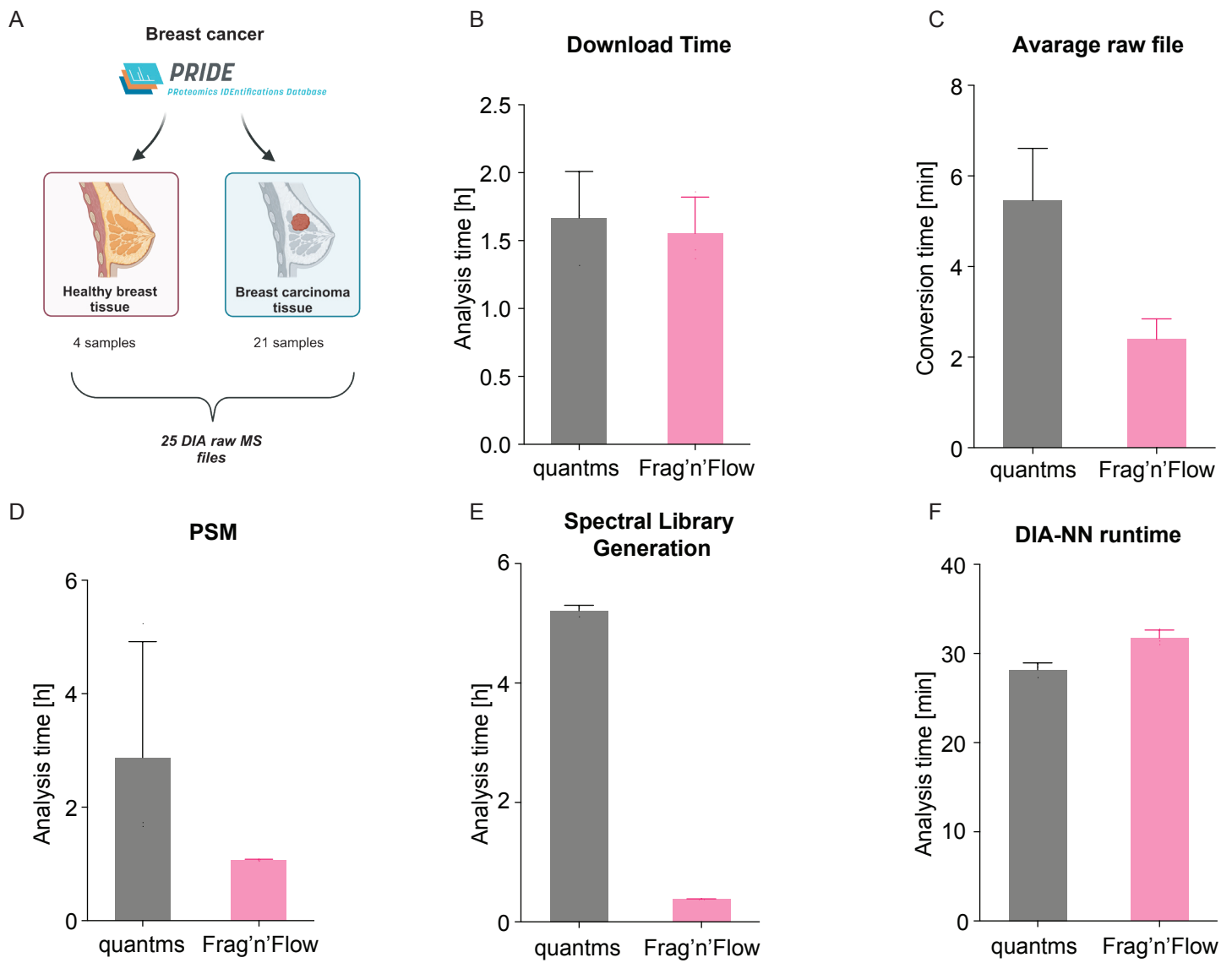

Supplementary Figure 4

Supplement: Supplementary file 7 — Additional file 7: Figure S4. Comparative analysis of quantms and Frag’n’Flow. A Schematics show the experimental design of the case study (DIA dataset) used for the benchmark. B Comparison of total download time for retrieving raw MS files from the PRIDE FTP server in hours. Error bars represent the standard deviation from three benchmark runs on the same dataset. C Average raw MS file conversion time per file during workflow execution in minutes. Error bars represent the standard deviation from three benchmark runs on the same dataset. D Comparison of total time needed for peptide spectrum in each of the workflows in hours. Error bars represent the standard deviation from three benchmark runs on the same dataset. E Comparison of total time needed for spectral library generation in hours. Error bars represent the standard deviation from three benchmark runs on the same dataset. F Comparison of total runtime of DIA-NN modules in both workflows in minutes. Error bars represent the standard deviation from three benchmark runs on the same dataset. [file 12859_2025_6305_MOESM7_ESM.pdf]
